# Explainable AI-driven MRI-based brain tumor classification: a novel deep learning approach

**DOI:** 10.3389/frai.2025.1700214

**Published:** 2026-01-08

**Authors:** Vinayaka R. Srinivas, Ramasubramanian Parvathi

**Affiliations:** School of Computer Science and Engineering, Vellore Institute of Technology, Chennai, India

**Keywords:** brain tumor classification, convolutional neural networks, data augmentation, deep learning, explainable AI, feature visualization, medical imaging, MRI

## Abstract

**Introduction:**

Brain tumors are among the most aggressive forms of cancer, requiring precise diagnosis and treatment planning to improve patient outcomes. This study aims to develop an efficient deep learning-based framework for the classification of brain tumors using MRI data.

**Methods:**

The methodology employs Convolutional Neural Networks (CNNs) to accurately classify tumors into four categories: normal, glioma, pituitary, and meningioma. Key preprocessing techniques, including noise reduction,resizing, and data augmentation, were applied to enhance the robustness of the model. Advanced architectures such as DenseNet50, VGG19, and other transfer learning models, along with CNN variants, were trained and evaluated for their performance. Explainable AI (XAI) techniques, including Grad-CAM, LIME, and feature map visualizations, played a crucial role in providing better visualizations of the model’s decision-making process and identifying areas of improvement during model training and to establish a better model.

**Results:**

The best-performing model, a 4-conv-1-dense-1-dropout CNN, achieved a classification accuracy of 95.86%, outperforming deeper architectures and transfer learning approaches. The findings underscore the potential of deep learning models for reliable and efficient brain tumor classification. This work concludes with recommendations for real-time deployment in clinical settings and explores future integration with Large Language Models (LLMs) to generate detailed diagnostic reports.

## 1 Introduction

With a mortality rate exceeding 80%, brain tumors are among the worst types of cancer. To improve the prognosis, a prompt and precise diagnosis is essential. However, the manual annotation and segmentation of a brain tumor can be a challenging task in medical analysis. Since each MRI modality offers a different set of information about the tumor locations, multiple modalities are typically evaluated. These MRI modalities tend to increase computation and overfitting, although they are useful for segmenting gliomas. This study presents a region of interest detection algorithm that may be used to identify important features and eliminate unnecessary MRI data during data pre-processing. As a result, the input size is reduced, enabling deeper neural networks and more aggressive data augmentations. Early detection of brain tumors is critical due to their rapid metastasis and growth.

Post-detection, the classification stage can be challenging and tedious for doctors or radiologists, especially in complex cases. This process heavily relies on the availability of expert medical personnel, which is often a luxury in underdeveloped and developing regions. The task involves specialists working on localizing the tumor, comparing it with adjacent tissues, applying necessary image enhancement techniques, and finally determining whether it could be a tumor and its type and grade. This fast and precise detection can be revolutionized through advancements in Artificial Intelligence, particularly in computer vision, image classification, and image segmentation, which have demonstrated high accuracy.

Deep learning, a subset of Machine Learning, utilizes neural networks that mimic the structure of the human brain and are trained with vast amounts of data. These systems, which can be supervised, semi-supervised, or unsupervised, show immense potential in medical image analysis. Comprising input, hidden, and output layers, deep learning algorithms use these multiple network layers for feature extraction and encoding. The output of each layer becomes the input for the next, aiding data abstraction as the network deepens. Artificial Neural Networks (ANN) and Convolutional Neural Networks (CNN) are popular in the industry, with CNNs particularly favored for image classification tasks due to their ability to select distinguishing features through convolving filters and pooling, followed by training the classification network’s layers.

## 2 Objective

The primary objective of using the Tumor dataset (Crystal Clean MRI dataset) is to develop machine learning models capable of accurately classifying and segmenting brain tumors from MRI images. This involves distinguishing between different types of brain tumors, such as gliomas, meningiomas, and pituitary tumors, or distinguishing between tumor and non-tumor cases. Early and accurate tumor classification is critical for effective diagnosis and treatment planning. To achieve this, the project aims to design a deep learning-based model, such as a Convolutional Neural Network (CNN), that significantly improves classification accuracy. The model will leverage techniques like data augmentation and preprocessing to enhance generalization, enabling it to perform effectively on real-world medical datasets. Moreover, exploring multimodal MRI data will further optimize classification performance, addressing the computational challenges posed by the high-dimensional nature of MRI scans.

In addition to improving accuracy, the project emphasizes reducing overfitting and optimizing computational efficiency. Overfitting, that limits a model’s ability to generalize to new data, will be mitigated using data augmentation, regularization methods, and cross-validation techniques. To enhance computational efficiency, strategies such as dimensionality reduction, region of interest (ROI) detection, and model pruning will be explored, minimizing memory usage and processing time while maintaining accuracy. The ultimate goal is to create a scalable and deployable solution for clinical use. The model will be designed to adapt to larger datasets, different MRI machines, or imaging centers. By being integrated into medical software or platforms used in hospitals, this solution could help healthcare professionals diagnose brain tumors more effectively and efficiently.

## 3 Related works

Role of deep learning in brain tumor detection and classification (2015 to 2020), Nazir et al. (2021) offers a comprehensive review of deep learning techniques, including CNN, RNN, and hybrid models, applied to brain tumor detection and classification between 2015 and 2020. However, it highlights a gap in exploring newer deep learning models post-2020 and addressing their real-time clinical applicability. This paper lays a foundation for understanding key methodologies but invites further research into cutting-edge models and practical applications.

An automated brain tumor classification in MR images using an enhanced convolutional neural network, Singh and Agarwal (2023) proposes an advanced CNN model that achieves high accuracy in classifying brain tumors using MRI images. The paper lacks an analysis of the model’s adaptability across different imaging modalities and real-time performance, suggesting that more investigation is needed into its versatility and speed for clinical application.

Machine learning in oncology: methods, applications, and challenges, Bertsimas and Wiberg (2020) provides a broad overview of machine learning methods applied in oncology, focusing on classification, segmentation, and treatment planning. Despite its breadth, the paper does not sufficiently emphasize brain tumors specifically and lacks a discussion on deep learning advancements, which could further enhance the field of oncology.

Multi-class brain tumor classification using residual network and global average pooling, Kumar et al. (2021) utilizes residual networks and global average pooling to classify brain tumors into multiple categories. While effective, the study does not adequately address the generalization of these models across diverse datasets and presents challenges with interpretability in clinical settings, leaving room for future research in these areas.

Comparative study of various techniques using deep learning for brain tumor detection, Gore and Deshpande (2020) compares multiple deep learning techniques for brain tumor detection, emphasizing accuracy and efficiency. However, the study does not provide a clear recommendation for the best-performing model and lacks clinical validation, making it a valuable comparison but limited in practical guidance for clinical applications Radiomics-based machine learning in differentiation between glioblastoma and metastatic brain tumors, Chen et al. (2019) utilizes radiomics and machine learning to differentiate glioblastoma from metastatic brain tumors. While promising, the study notes challenges in scaling the model across diverse patient data and different MRI devices, highlighting the need for more generalizable models.

Brain tumor classification using convolutional neural network, Abiwinanda et al. (2019) demonstrates a CNN-based approach for brain tumor classification, emphasizing simplicity and accuracy. However, it does not explore more complex deep learning architectures, such as transformers or hybrid models, suggesting that future research could investigate the potential benefits of more advanced architectures.

Classification of brain tumors and auto-immune disease using ensemble learning, Shafi et al. (2021) uses ensemble learning to classify brain tumors and autoimmune diseases, achieving enhanced predictive performance. However, the study lacks evaluation across various demographic groups and does not include external validation, which could improve the model’s robustness and generalization.

Brain tumor detection: a long short-term memory (LSTM)-based learning, odel Amin et al. (2020) introduces an LSTM-based model aimed at enhancing brain tumor detection performance. Despite its novel approach, the paper provides limited comparisons with other sequential models like GRUs and lacks performance testing on larger datasets, suggesting areas for expanded exploration.

Multimodal brain tumor classification using deep learning and robust feature selection, Khan et al. (2020) applies a multimodal approach that combines imaging and non-imaging data, using robust feature selection for classification. However, challenges arise in the real-time acquisition of multimodal data and computational efficiency, indicating that future research could focus on optimizing these areas for practical application.

Brain MRI Classification and Segmentation of Glioma, Pituitary, and Meningioma Tumors Using Deep Learning Approaches, Mostafa et al. (2024) focuses on the segmentation and classification of different brain tumor types using CNNs and advanced deep learning methods. Yet, the study offers limited exploration of how segmentation errors may impact classification accuracy, leaving room for more investigation into these interconnected processes.

A sequential machine learning-cum-attention mechanism for effective segmentation of brain tumor, Ali et al. (2022) presents a sequential machine learning model with an attention mechanism designed to improve segmentation accuracy. However, the study does not address issues related to clinical integration and scalability, which could hinder its practical application in healthcare settings.

Brain tumor segmentation using deep capsule network and latent-dynamic conditional random fields, Elmezain et al. (2022) proposes a deep capsule network with latent-dynamic conditional random fields for accurate segmentation. The paper highlights the complexity of model training and notes challenges with interpretability in medical environments, indicating areas for future research to simplify and explain these models.

Brain tumors classification for MR images based on attention-guided deep learning model, Zhang et al. (2021) employs attention-guided deep learning models to improve classification accuracy in MRI-based brain tumor detection. The study lacks a thorough discussion on the robustness and interpretability of the model in clinical settings, highlighting areas for improvement in clinical applicability.

A Robust Deep Learning Model for Brain Tumor Detection and Classification Using EfficientNet: A Brief Meta-Analysis (Singh et al., 2024), leverages EfficientNet for brain tumor detection and classification, supported by a meta-analysis of its performance. However, the paper does not address the real-time performance of EfficientNet or its computational requirements, suggesting potential areas for further investigation in practical deployment.

Design of encoded graphene-gold metasurface-based circular ring and square sensors for brain tumor detection and optimization using XGBoost algorithm, Patel et al. (2024) introduces novel metasurface-based sensors, optimized with the XGBoost algorithm, for brain tumor detection. While innovative, the study points out challenges with scaling this method for widespread clinical use and lacks a clear pathway for clinical integration, leaving room for future studies on feasibility and scalability.

Personalized treatment planning and predicted therapy response based on different MRI data sets, classified the tumor tissue type, identified the severity of the tumor were discussed in Missaoui et al. (2025).

Recent studies such as BrAInVision, Gagliardi et al. (2025) have demonstrated that hybrid and doubly explainable architectures—combining handcrafted and deep features—can enhance both accuracy and interpretability in brain MRI classification. Similarly, Missaoui et al. (2025) emphasized the integration of advanced transformer-based and hybrid CNN models, highlighting challenges in computational scalability and transparency that our lightweight CNN addresses.

Various techniques used for transparency, logical and ethical dimensions of AI decision justification applied to the clinical contexts are explained in Caroprese et al. (2022).

Importance of data privacy in medical imaging and highlighted how federated learning enables distributed model training while safeguarding patient confidentiality was discussed in Caroprese et al. (2023).

## 4 Dataset

The Crystal Clean Brain Tumor MRI Dataset is a comprehensive collection of high-resolution MRI scans designed to support precise brain tumor classification. The dataset consists of detailed MRI images that capture intricate brain structures, making it invaluable for distinguishing subtle features across various tumor types. The Crystal Clean Brain Tumor MRI dataset used in this study was obtained from a publicly available Kaggle repository.^1^ The dataset contains T1-weighted contrast-enhanced MRI scans categorized into four classes: glioma, meningioma, pituitary, and normal. Initially, the dataset comprised 3,264 images—826 glioma, 822 meningioma, 830 pituitary, and 786 normal images. Following data augmentation (including rotation, flipping, and brightness variation), the dataset expanded to 13,056 images to balance class distribution and improve model generalization. The data were split into 80% for training and 20% for testing, ensuring class balance across both subsets.

**Figure 1:**
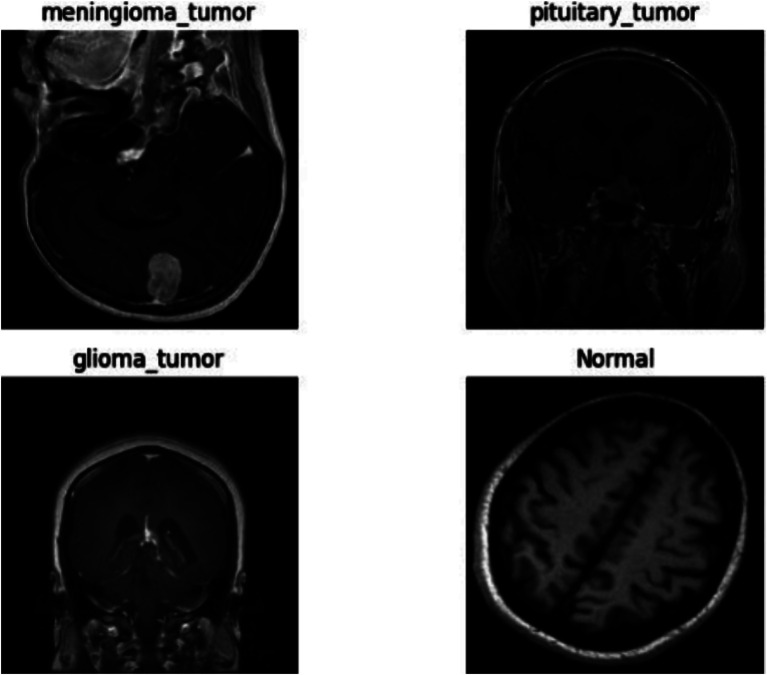
Different types of datasets images.

Each image is meticulously annotated to indicate the presence or absence of a tumor (refer Figure 1), with tumor types labeled as glioma, meningioma, pituitary tumor, or no tumor cases. These high-quality images, paired with accurate annotations, serve as a robust foundation for training machine learning models, enabling researchers to achieve precise tumor identification and classification.

## 5 Methodology

### 5.1 Preprocessing

While the Crystal Clean MRI dataset was preprocessed by the provider, additional refinement steps were performed to enhance image consistency and model readiness. These included verifying tumor region integrity, re-cropping based on the largest visible contour, resizing to 224 × 224 pixels, and applying normalization. These additional steps ensured uniformity across samples and removed residual background noise. In the preprocessing stage, efforts were made to ensure uniformity and quality in the dataset before feeding it into neural networks. The preprocessing steps, including resizing, normalization, and formatting of the image data, were already performed by the dataset provider. We are grateful for the well-prepared dataset, which allowed us to focus on model development and evaluation without requiring additional pre-processing efforts. High-resolution MRI images were resized to a standard dimension of 224x224x3, preserving critical information while reducing storage requirements. Since the black background surrounding the brain in MRI images does not contribute meaningful data for classification, the images were cropped to remove this irrelevant region.

However, in the pre-processing stage, we attempt to make uniformity in data before feeding it to neural networks. Our images had high resolution, and we scaled them back to 224x224x3, which helps preserve all relevant data while reducing storage requirements. The MRIs contained a black background around the central image of the brain. This dark background gives no valuable data for classification since no real information of the MRI is present in the dark background. Subsequently, the images were trimmed around the main contour. Here, the greatest contour is chosen and marked. Following, we discover the extreme points of the contour and crop the image on those endpoints. Thus, removing most of the background and noise present within the original image. This process is done for each image in the dataset. Images which do not adhere to the conditions are discarded from the classification process. Such images come about in distorted shapes and were removed by manual review.

- Removal of Duplicate Samples: We employed an image vector comparison method to identify and remove duplicate samples, ensuring that each data point is unique.
- Correction of Mislabeled Images: Using our domain knowledge, we carefully inspected and corrected falsely labeled images, ensuring that they were appropriately categorized. This step greatly enhances the accuracy of the dataset.
- Image Resizing: All images in the dataset were resized to a memory-efficient yet academically accepted size of (224, 224), facilitating easier processing and analysis.

**Figure 2:**
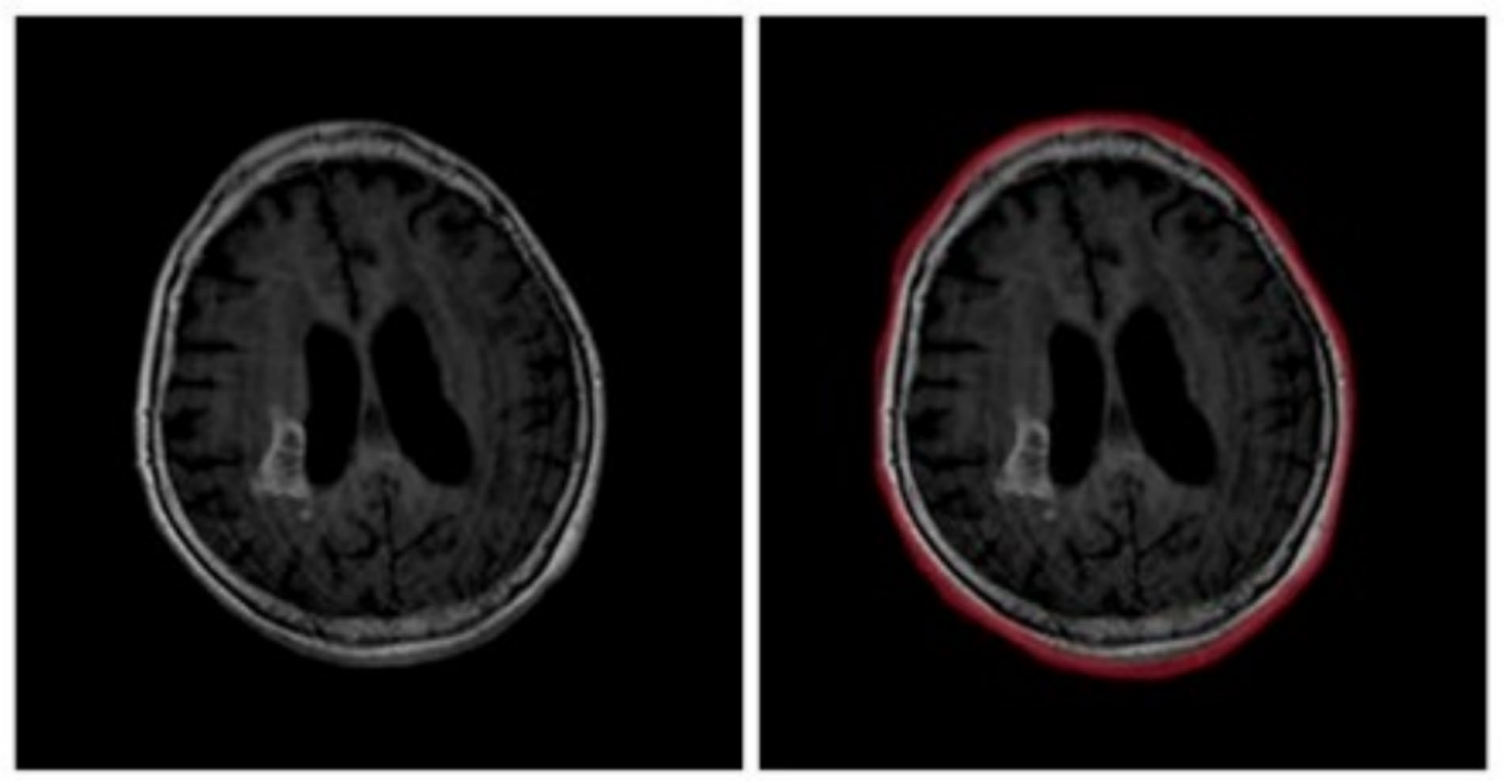
Tumor contour of MRI images.

This was achieved by identifying the largest contour in each image, determining its extreme points, and cropping accordingly (refer Figure 2), effectively isolating the brain region while minimizing background noise. Any distorted or non-conforming images were manually reviewed and excluded from the dataset. Further preprocessing steps included the removal of duplicate samples through image vector comparisons, ensuring that each data point was unique. Additionally, mislabelled images were identified and corrected using domain expertise to improve labelling accuracy and improve dataset reliability.

### 5.2 Augmentation

**Figure 3:**
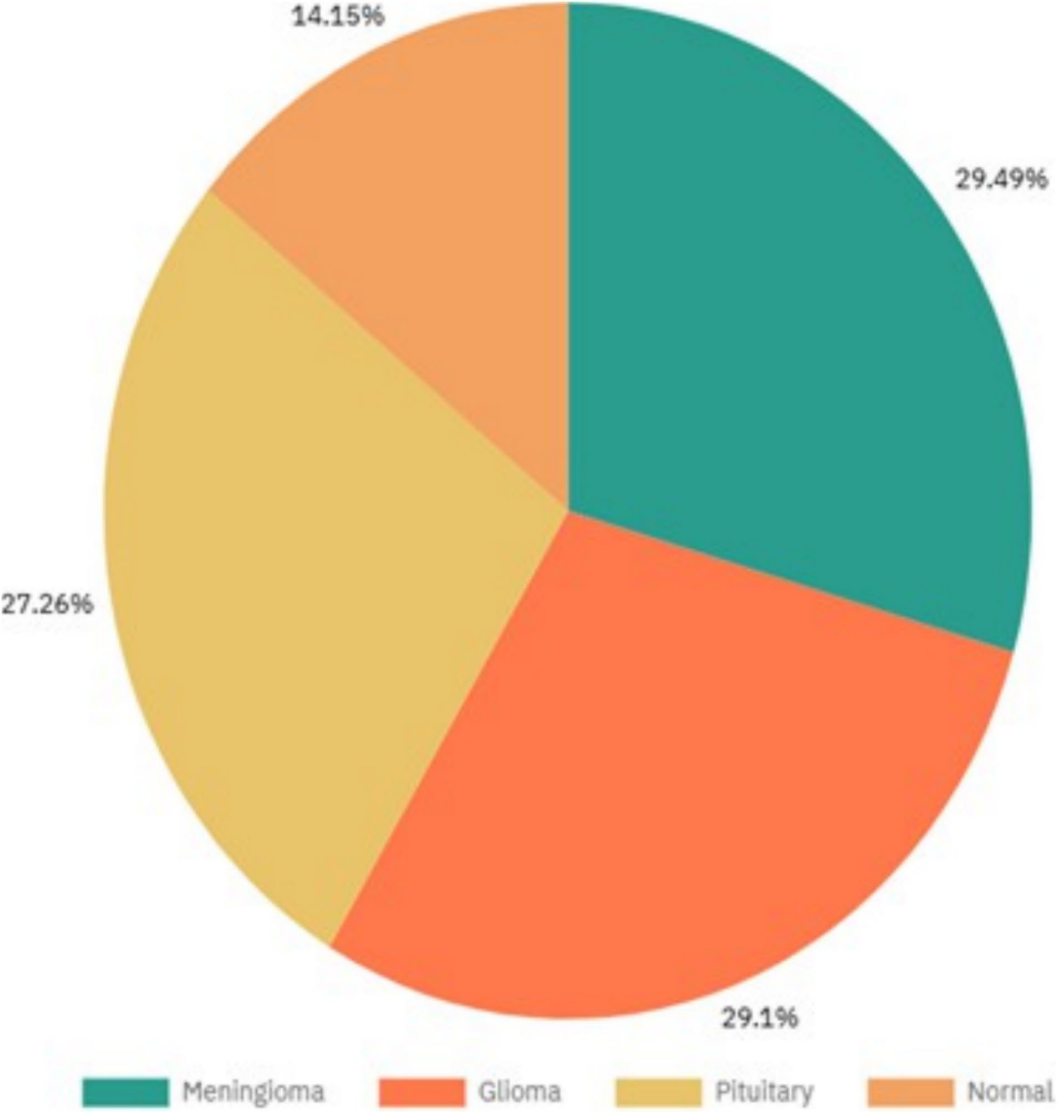
Depicts the percentage of images in each class.

To enhance the robustness of the dataset and improve the model’s ability to generalize, several data augmentation techniques were employed. The distribution of data across the four classes—Normal, Glioma, Pituitary, and Meningioma—is shown in Figure 3.

Salt and Pepper Noise introduces random noise to the images by setting pixels to white (salt) or black (pepper) at specific intensities. This technique simulates real-world imperfections, such as sensor noise or transmission errors, which often occur in medical imaging. By exposing the model to noisy data, it becomes more resilient in handling distorted or degraded images, thereby improving its performance in practical scenarios.

Histogram Equalization was applied to improve image contrast by redistributing pixel intensity values. This method enhances subtle details in images that may appear washed out or underexposed. For MRI scans, this is crucial as it brings out intricate patterns and features, enabling the model to better differentiate between tumor and non-tumor regions.

Rotation involved altering the orientation of images by rotating them clockwise or counterclockwise. This augmentation accounts for the slight variations in orientation that can occur during MRI scans. By diversifying the dataset with rotated images, the model learns to identify tumors regardless of the alignment of the image, improving its adaptability to real-world data.

**Figure 4:**
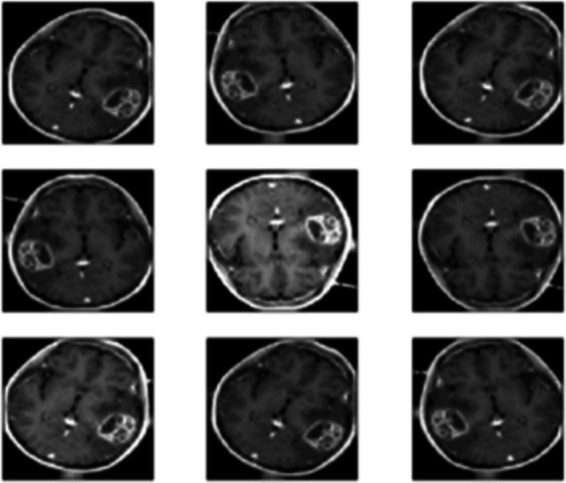
Augmentation of MRI images.

Brightness Adjustment simulated varying lighting conditions by increasing or decreasing the image intensity values. Since MRI images might differ in brightness due to variations in equipment or imaging protocols, this technique ensures the model can handle such discrepancies. Training on images with varied brightness levels improves the model’s ability to make accurate classifications under diverse imaging conditions. Lastly, Horizontal and Vertical Flipping created mirror images of the data, effectively doubling the dataset size and introducing spatial orientation variability. For symmetrical structures like the brain, flipping helps the model recognize tumors regardless of their location within the brain’s symmetry as seen in Figure 4. This augmentation increases data diversity and helps the model generalize better between different spatial configurations. This constitutes a large dataset of 22,000 MRI images which is used for the training process.

### 5.3 Justification for using CNNs, transfer learning, and explainable AI

The choice of CNN-based models and transfer learning architectures over more complex state-of-the-art models was driven by practical considerations such as hardware limitations, computational efficiency, and accessibility for smaller research environments or medical institutions with limited resources. While transformer-based architectures and advanced deep learning models like Vision Transformers (ViTs) or hybrid networks have shown promising results in medical imaging, they demand extensive computational power, large-scale labelled datasets, and specialized hardware such as high-end GPUs or TPUs. Many small-scale healthcare facilities, startups, and research groups lack the infrastructure to deploy such resource-intensive models in real-world clinical applications.

CNNs, on the other hand, provide a highly efficient alternative—they require significantly lower computational resources while still achieving high accuracy, making them more feasible for on-device processing, cloud deployment, or integration into hospital imaging systems. Additionally, transfer learning with models like VGG19 and DenseNet50 allows leveraging pre-trained feature extraction while minimizing training time and data requirements, a crucial advantage when working with medical datasets that are often limited in size. In this study, we use transfer learning methods not only to compare their results against CNN models but also to demonstrate the effectiveness of custom CNN architectures for the classification of brain tumors. By showcasing performance differences, we highlight how CNNs offer a more practical approach for small-scale projects and resource-limited environments, ensuring accessibility without compromising accuracy.

The explainability aspect of the chosen CNN model also enhances its practical applicability compared to other complex architectures. Techniques such as Grad-CAM and LIME provide interpretable visualizations that highlight the model decision-making process, strengthening trust in clinical environments, and helping create an architecture of significant results. Unlike models such as Xception and InceptionV3, which exhibit higher computational complexity with marginal accuracy gains, the proposed CNN model remains computationally efficient, making it suitable for real-time deployment in medical diagnostics. Furthermore, transfer learning models require extensive fine-tuning to adapt to medical datasets, whereas the proposed CNN, trained from scratch on domain-specific data, achieves high accuracy with tailored feature representation.

### 5.4 Architecture diagram

**Figure 5:**
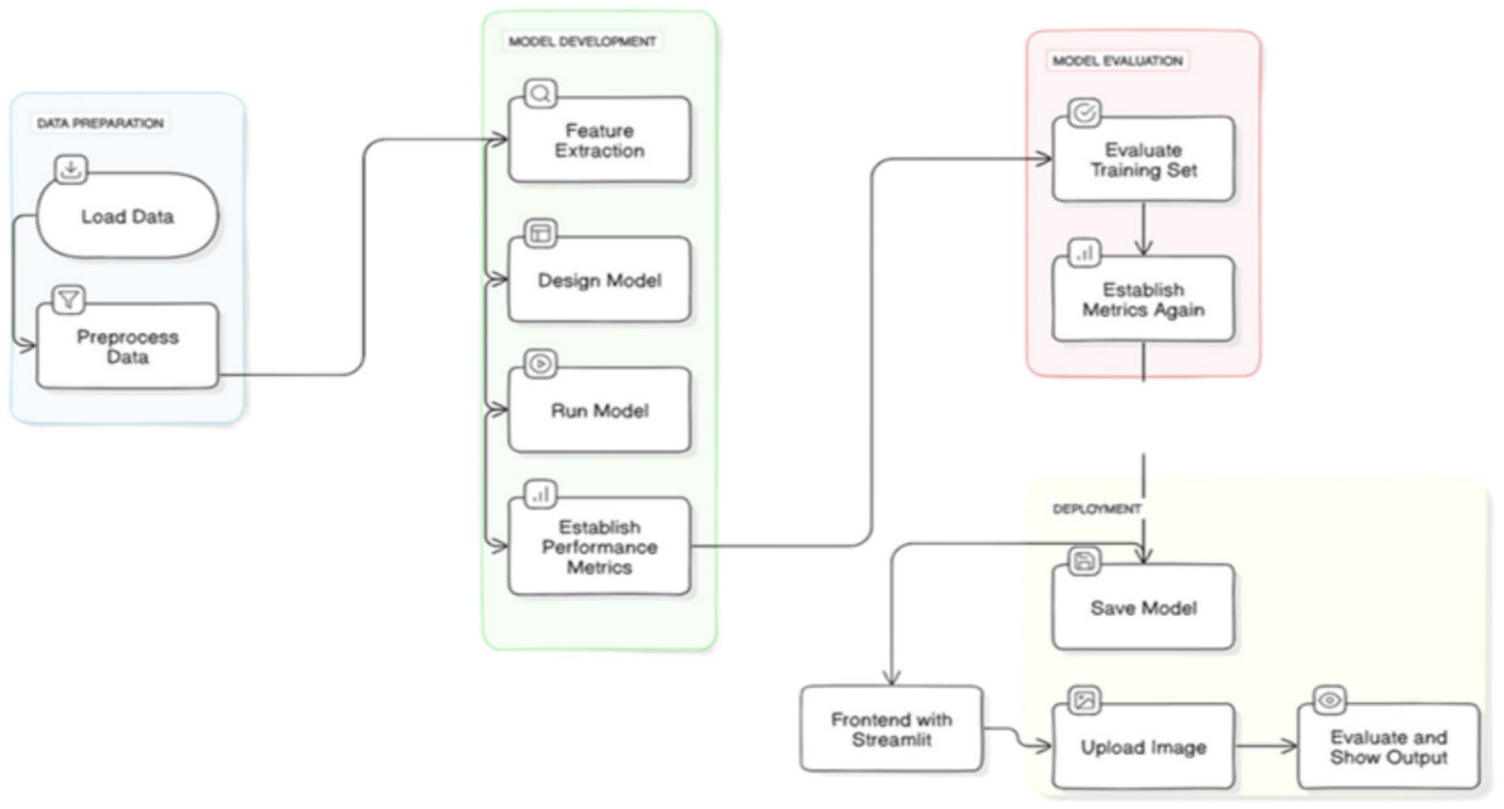
Overall architecture for brain tumor classification.

The overall architecture of the proposed brain tumor classification is illustrated in Figure 5.

#### 5.4.1 Data preparation

Load Data: The first step is loading the MRI images needed for tumor classification. This data forms the basis for training and testing the model.

Preprocess Data: After loading, the data is preprocessed to ensure it is clean and consistent. Common preprocessing steps might include resizing images, normalizing pixel values, removing noise, and data augmentation techniques to increase the dataset’s diversity and robustness. Proper preprocessing is essential to improve model accuracy and performance.

#### 5.4.2 Model development

Feature Extraction: After the data is prepared, it is followed by feature extraction, where meaningful characteristics or patterns are extracted from the images. For MRI data, features could include pixel intensities, textures, shapes, or tumor-specific markers. These features help the model distinguish between different types of tumors or between tumor and non-tumor cases.

Design Model: In this step, a deep learning or machine learning model architecture is chosen and designed based on the project requirements. Common architectures for image classification tasks include Convolutional Neural Networks (CNNs) or other neural network models optimized for visual data processing.

Run Model: After designing the model, it is trained on the preprocessed dataset. During this phase, the model learns from the features to differentiate between tumor types or identify the absence of a tumor. Training involves adjusting model parameters to minimize errors and improve classification accuracy.

Establish Performance Metrics: As the model trains, performance metrics such as accuracy, precision, recall, F1 score, or AUC (Area Under the Curve) are calculated to evaluate the model’s effectiveness. These metrics help assess whether the model is learning effectively and can generalize well to new, unseen data.

#### 5.4.3 Model evaluation

Evaluate Training Set: After completing the training, the model is assessed on the training set to determine if it is overfitting or underfitting. Overfitting happens when the model excels on the training data but fails on new data, whereas underfitting indicates that the model is not effectively identifying the underlying patterns in the data.

Establish Metrics: After evaluating on the training set, performance metrics are recalculated to ensure consistency and validate the model’s effectiveness. This step might also include testing the model on a separate validation or test dataset to gauge its real-world applicability.

#### 5.4.4 Deployment

Save Model: Once the model performs satisfactorily, it is saved for deployment. This saved model can then be loaded in a production environment where it can be used for real-time tumor classification.

Frontend with Streamlit: A Streamlit interface is designed to allow users to interact with the model easily. Streamlit is a popular framework for creating web applications for machine learning models, making it simple to create a user-friendly frontend.

Upload Image: In the deployed system, users can upload MRI images through the Streamlit interface. These images are then passed to the model for classification.

Evaluate and Show Output: Finally, the model processes the uploaded image, performs the tumor classification, and returns the result to the user. The result, including predicted tumor type and any other relevant metrics or visualizations, is displayed on the frontend for easy interpretation.

### 5.5 Convolution neural networks

The proposed CNN model of 3-conv-128-nodes-2-dense-2 dropout demonstrates superior performance in brain tumor classification by balancing precision, generalization, and computational efficiency. Unlike shallower architectures such as 3-conv-64-nodes-3-dense which exhibited lower validation accuracies due to insufficient feature extraction, the selected model optimally captures tumor-specific patterns while mitigating overfitting through L2 regularization and dropout. The experimental results confirm that this model achieves a validation accuracy of 92.76%, outperforming deeper architectures such as 5-conv-128-nodes-3-dense-2-dropout, which, despite achieving 91.96%, showed marginally higher validation loss, suggesting potential overfitting.

However, a more refined architecture, the 4-conv-128-nodes-1-dense-1-dropout CNN model, emerges as the best-performing among all evaluated configurations. This model achieves an impressive accuracy of 95.86% with a validation accuracy of 95.32%, along with a training loss of 0.1232 and a validation loss of 0.1557. These results indicate a well-generalized model with improved feature extraction capabilities while maintaining robustness against overfitting. In contrast, the 3-conv-128-nodes-2-dense-2-dropout model, though effective, attained a relatively lower accuracy of 92.88% with a higher validation loss of 0.26, reinforcing the advantage of deeper feature extraction in the 4-conv-128 architecture.

#### 5.5.1 4-conv-1-dense-1-dropout architecture

The 4-conv-128-nodes-1-dense-1-dropout architecture described in Algorithm 1 demonstrated the highest performance among the evaluated CNN models for brain tumor classification. With a training accuracy of 95.86% and a validation accuracy of 95.32%, this model achieved superior generalization while maintaining a low validation loss of 0.1557.

Compared to other architectures, such as the 3-conv-128-nodes-2-dense-2-dropout model, which reached a validation accuracy of 92.76%, the additional convolutional layer in this design effectively captured intricate tumor-specific features without introducing significant overfitting. The balanced depth of the architecture, coupled with a single dropout layer, contributed to its robustness, ensuring that the model retained sufficient feature extraction capacity while mitigating excessive regularization effects that could hinder performance.

**Figure 6:**
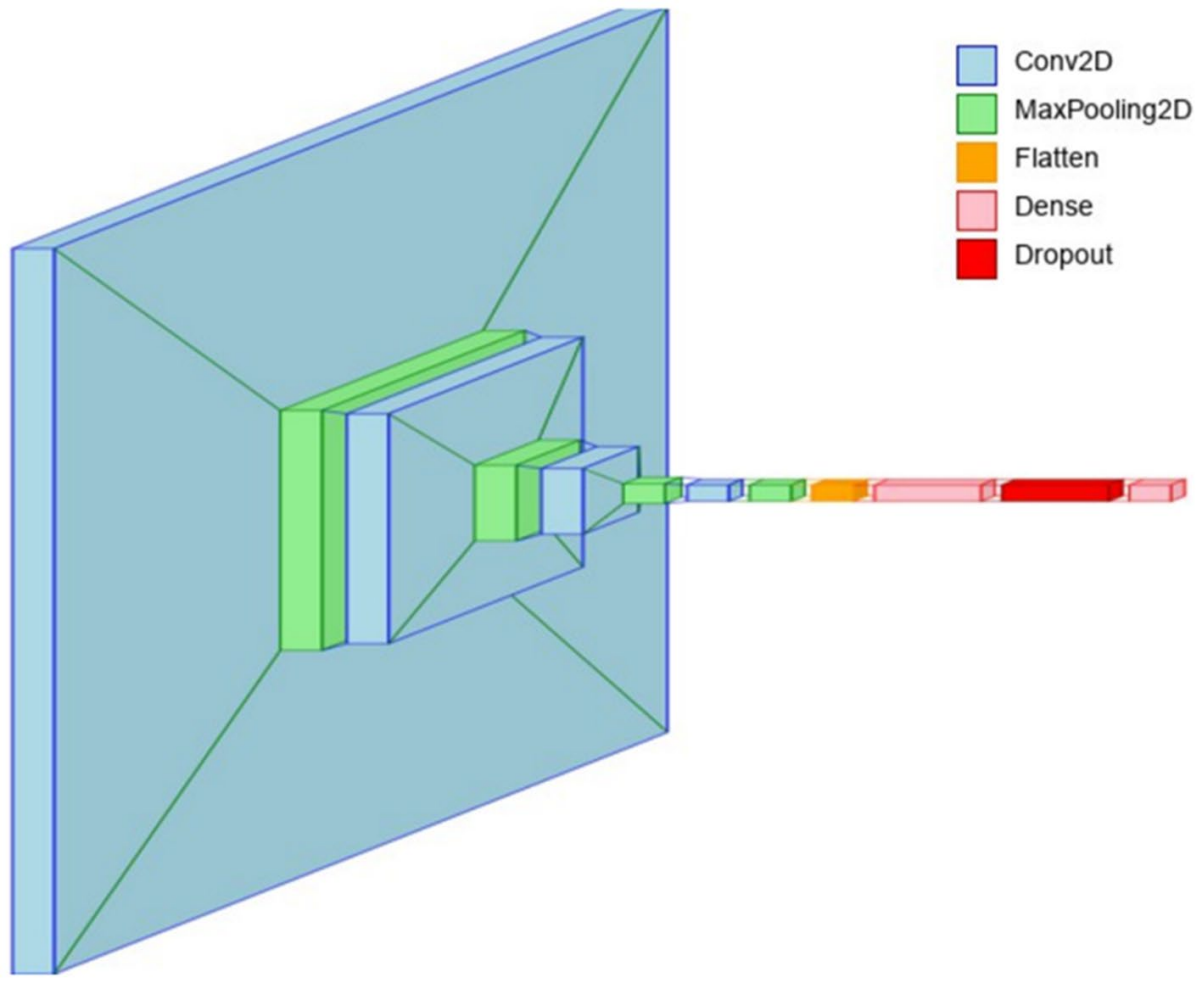
Layer view of the 4-conv-1-dense-1-dropout architecture.

The 4-conv-128-nodes-1-dense-1-dropout architecture (see Figure 6) is designed to achieve a balance between deep feature extraction and computational efficiency for brain tumor classification. The first convolutional layer, consisting of 32 filters with a 4 × 4 kernel size and ReLU activation, captures fundamental spatial patterns such as edges and textures. These low-level features are then refined by the second convolutional layer, which increases the filter count to 64, enabling the model to learn more complex structures. Max pooling follows each convolutional layer, reducing the spatial dimensions while retaining essential feature representations. The third and fourth convolutional layers, both equipped with 128 filters, enhance the network’s ability to detect intricate tumor-specific patterns, allowing the model to differentiate between.

ALGORITHM 1 4-conv-1-dense-1-dropout (L2 regularization) architecture**Require:**
MRI input images of size (224, 224, 3)
**Ensure:** Output: Class label (Normal, Glioma, Pituitary, Meningioma)
Initialize a sequential CNN model
Add Conv2D layer with 32 filters, kernel size (3,3), ReLU activation
Add MaxPooling2D layer
Add Conv2D layer with 64 filters, ReLU activation, L2 regularization
Add MaxPooling2D layer
Add Conv2D layer with 128 filters, ReLU activation, L2 regularization
Add MaxPooling2D layer
Add Conv2D layer with 128 filters, ReLU activation, L2 regularization
Add MaxPooling2D layer
Flatten feature maps
Add Dense layer with 512 units, ReLU activation
Add Dropout layer (rate: 0.5)
Add Output Dense layer with 4 units, Softmax activation
Compile using Adam optimizer and Categorical Crossentropy loss
Train model and evaluate using Accuracy, Precision, Recall, and F1-Score

After the convolutional feature extraction process, the output is flattened and passed through a 512-node fully connected layer, where learned features are aggregated for classification. The inclusion of a single dropout layer (50%) prevents overfitting by randomly deactivating neurons during training, ensuring better generalization to unseen medical images. The softmax activation in the final dense layer facilitates multi-class tumor classification. To enhance model interpretability, Grad-CAM (Gradient-weighted Class Activation Mapping) and LIME (Local Interpretable Model-agnostic Explanations) were employed. Grad-CAM visualizations provided heatmaps that highlighted the most influential regions in MRI scans, ensuring that the convolutional layers were focusing on tumor regions rather than background artifacts. LIME further validated the model’s decision-making by generating local perturbations and identifying key image regions that contributed to the final classification. These explainability techniques reinforce the model’s clinical applicability, ensuring transparency in decision-making for medical professionals.

**Figure 7:**
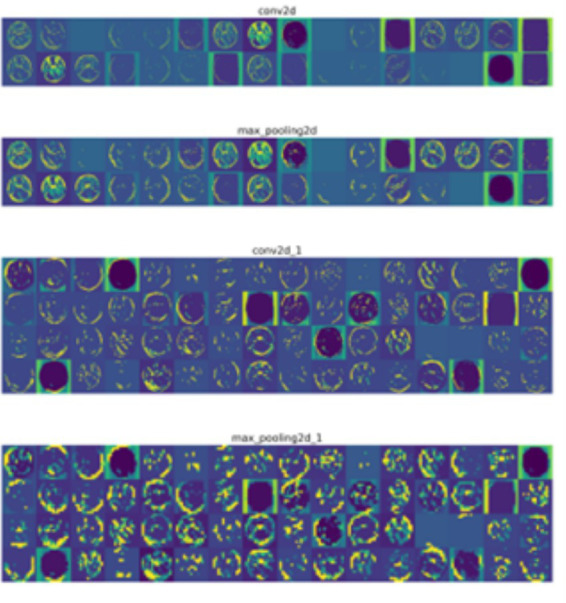
Visualizing convolution layer activations of 4-conv-1-dense-1-dropout architecture.

The activation maps (see Figure 7) provide a detailed visualization of how different layers of the 4-conv-1-dense-1-dropout architecture respond to input images. The convolutional layers exhibit strong activations in specific regions, highlighting the model’s ability to capture intricate spatial patterns such as edges, textures, and fine details. The max-pooling layers compress the spatial representation, reducing dimensionality while preserving the most significant features. By analyzing these activation maps, critical insights were obtained regarding the model’s focus areas and its generalization to different inputs. In conjunction with Grad-CAM and LIME visualizations, these activation maps played a pivotal role in refining hyperparameters, adjusting dropout rates, and modifying convolutional filter sizes to enhance the model’s performance. Through iterative modifications based on these visualizations, the final model was optimized to achieve robust feature extraction and improved classification accuracy, ensuring a well-adapted architecture for the dataset.

Furthermore, this architecture outperformed deeper models such as the 5-conv-128-nodes-3-dense-2-dropout, which, despite achieving a competitive validation accuracy of 91.96%, exhibited a marginally higher validation loss, indicating potential overfitting. Similarly, shallower models like the 3-conv-64-nodes-2-dense-2-dropout, which reached a validation accuracy of 92.76%, failed to extract the necessary high-level spatial features required for precise tumor classification. These results highlight that the 4-conv-128-nodes-1-dense-1-dropout configuration achieves an optimal trade-off between accuracy, computational efficiency, and model complexity, making it the most suitable architecture for this task. This balance is particularly crucial in real-world medical applications, where maintaining high diagnostic accuracy with minimal resource utilization is essential for deployment in clinical settings.

#### 5.5.2 3-conv-2-dense-2-dropout(L2 regularization) architecture

This model described in Algorithm 2 effectively avoids both overfitting and underfitting, allowing it to generalize well on the test set while accurately learning key features from the training data. The architecture includes three convolutional layers that are responsible for extracting important features from the input images, followed by two dense (fully connected) layers that interpret these features for final predictions. The inclusion of two dropout layers reduces overfitting by randomly dropping a fraction of neurons during training, encouraging the model to learn more generalized features.

**Figure 8:**
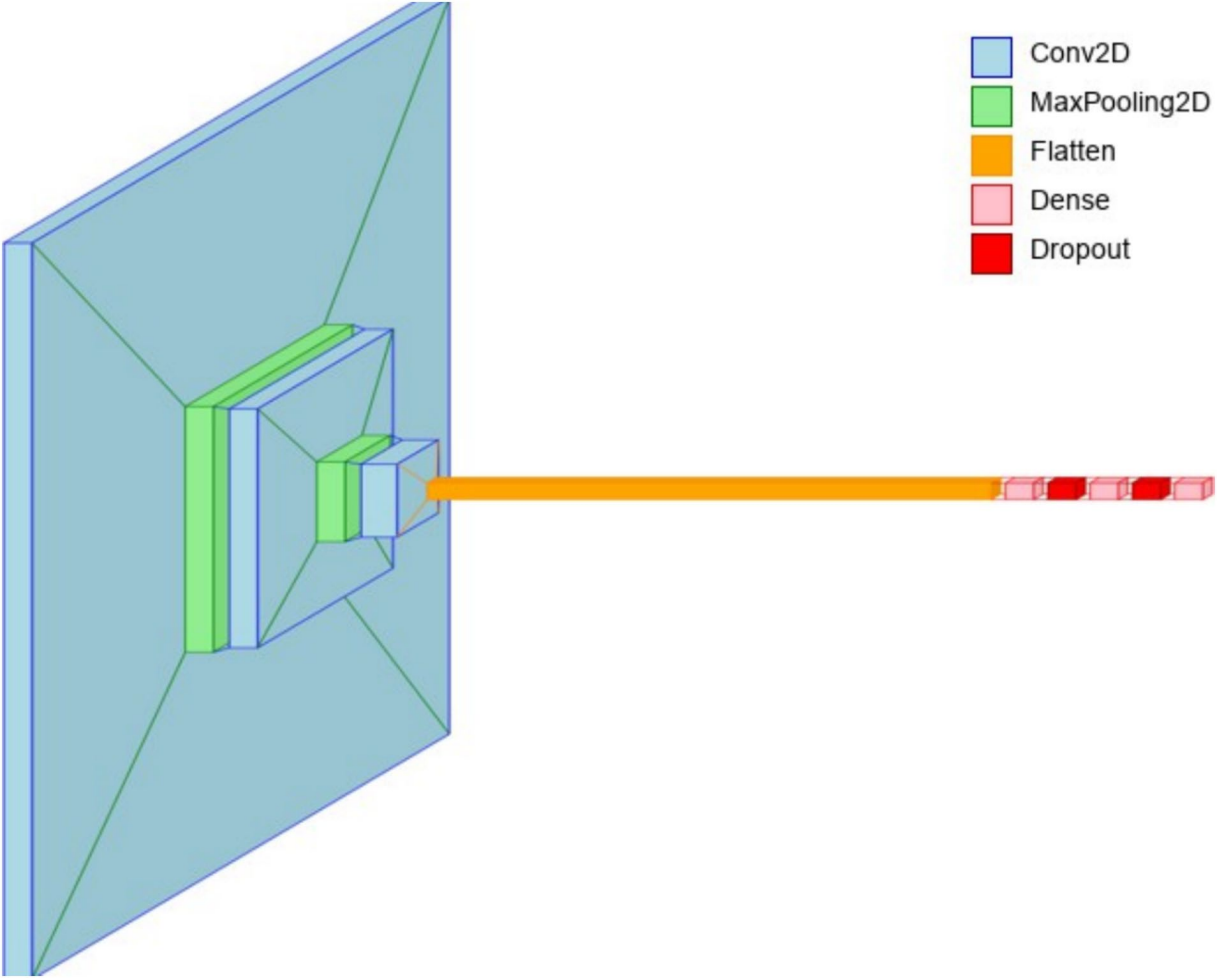
Layer view of the 3-conv-2-dense-2-dropout architecture.

The architecture of 3-conv-128-nodes-2-dense-2-dropout (see Figure 8) effectively balances feature extraction, computational efficiency, and generalization for brain tumor classification. The first convolutional layer, utilizing a 3 × 3 kernel with 64 filters and ReLU activation, captures low-level features such as edges, textures, and gradients. This fundamental feature extraction allows subsequent layers to build upon simple patterns to detect more complex structures. The second convolutional layer, also with 64 filters, enhances pattern recognition by identifying contours and higher-order features, further refined through max pooling to reduce spatial dimensions. The third convolutional layer, with 128 filters and L2 regularization, expands feature learning, ensuring deeper pattern extraction while mitigating overfitting. This structured feature extraction process enables the model to learn tumor-specific characteristics efficiently.

This architecture with L2 regularization is considered the optimal model due to its balanced performance. This model effectively avoids both overfitting and underfitting, ensuring that it generalizes well on the test set while still learning the key features of the training data. The architecture consists of five convolutional layers, which help extract increasingly complex features from the input images, followed by a series of fully connected dense layers that interpret these features to make predictions. The use of two dropout layers helps mitigate overfitting by randomly dropping a fraction of the neurons during training, forcing the model to learn more robust features. Additionally, L2 regularization is applied to the convolutional layers, which helps prevent the model from becoming too complex and overfitting by penalizing large weights.

ALGORITHM 2 3-conv-2-dense-2-dropout architecture **Require:** MRI input images of size (224, 224, 3)
**Ensure:** Output: Class label (Normal, Glioma, Pituitary, Meningioma)
Initialize a sequential CNN model
Add Conv2D layer with 64 filters, ReLU activation, L2 regularization
Add MaxPooling2D layer
Add Conv2D layer with 128 filters, ReLU activation, L2 regularization
Add MaxPooling2D layer
Add Conv2D layer with 256 filters, ReLU activation, L2 regularization
Add MaxPooling2D layer
Flatten the output
Add Dense layer with 512 units, ReLU activation
Add Dropout layer (rate = 0.4)
Add Dense layer with 256 units, ReLU activation
Add Dropout layer (rate = 0.4)
Add Output Dense layer with 4 units, Softmax activation
Compile model with Adam optimizer and Categorical Crossentropy loss
Train and validate using stratified data split, evaluate with class-wise metrics

Following convolutional processing, the extracted feature maps are flattened and passed through two fully connected dense layers. The first dense layer consists of 64 neurons, integrating information from previous layers, while the second dense layer functions as the output layer, classifying images into one of the four tumor categories via softmax activation. The incorporation of dropout layers (40%) significantly reduces overfitting by randomly deactivating neurons during training, ensuring robust generalization. Activation visualizations confirm that the network effectively learns relevant tumor features, with high activations corresponding with critical tumor regions. The integration of Grad-CAM and LIME further validated the model’s reliability, revealing that key areas influencing classification align with known tumor structures. This transparency reinforces the model’s clinical applicability, ensuring it focuses on meaningful tumor regions rather than irrelevant image artifacts. Grad-CAM visualizations provided heatmaps that highlighted the most influential regions in MRI scans, ensuring that the convolutional layers were correctly focusing on tumor regions rather than background artifacts.

#### 5.5.3 5-conv-3-dense-2-dropout architecture

The 5-conv-128-nodes-3-dense-2-dropout architecture described in Algorithm 3 follows a structured feature extraction process, progressively learning low-level, mid-level, and high-level patterns from MRI images.

**Figure 9:**
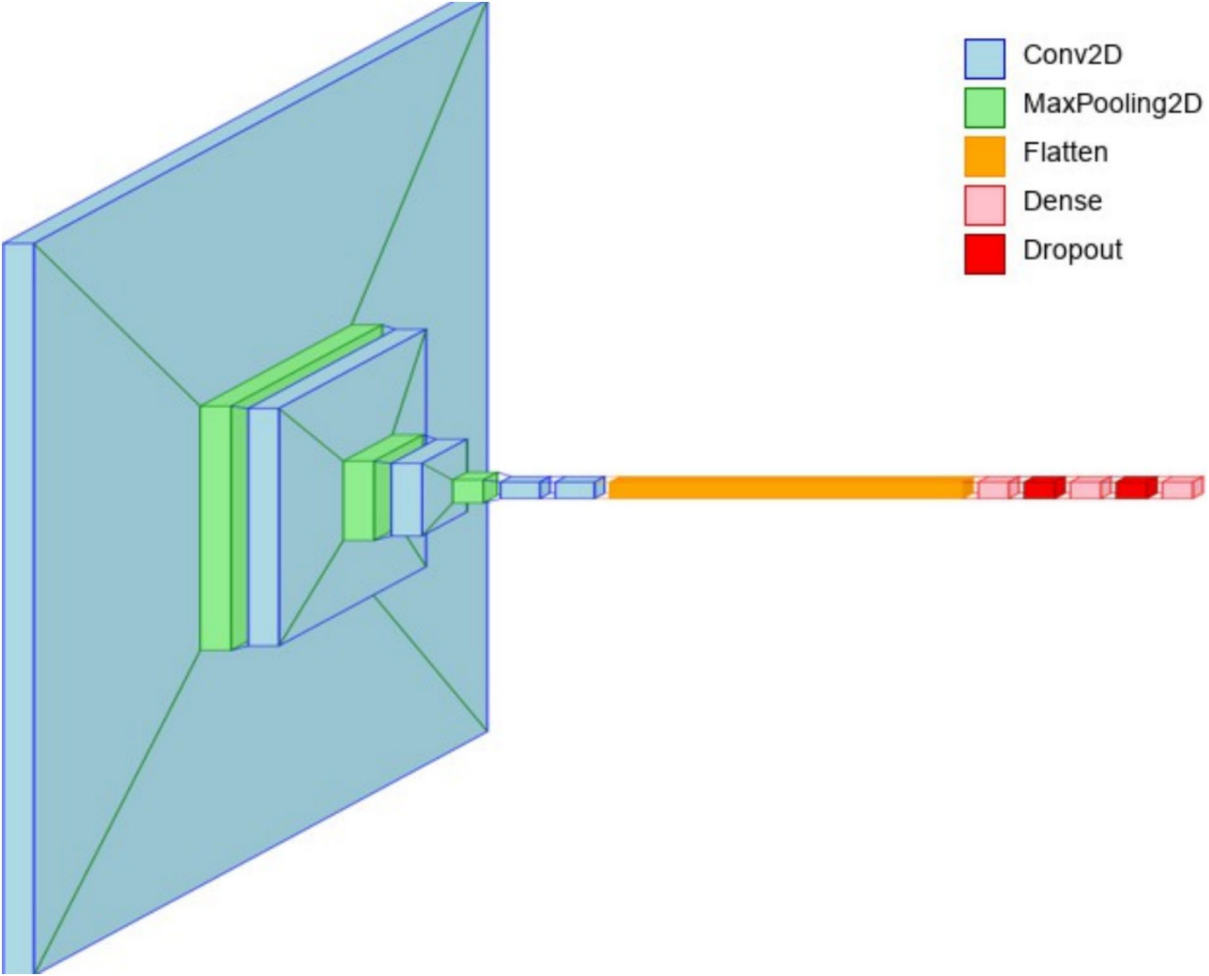
Layer view of the 5-conv-3-dense-2-dropout architecture.

These layers exhibit high activation across multiple filters, indicating that they extract fine-grained structural details. As the network progresses to mid-level convolutional layers (see Figure 9), activation becomes more selective, focusing on region-specific patterns that distinguish tumor structures from normal tissue. This reduction in activation density implies that the network is filtering out less relevant information, concentrating on important tumor characteristics. In the final convolutional layers, the activations become even more sparse and localized, signifying the network’s ability to abstract complex tumor-specific representations while discarding irrelevant background features. This hierarchical feature refinement process enables robust classification with minimal computational redundancy.

After convolutional feature extraction, the dense layers integrate the learned representations to form the final classification decision. The first two dense layers process a diverse set of activation values, selectively amplifying features critical to distinguishing tumor types. The visualized activations in these layers reveal a broad range of responses, highlighting their role in refining tumor-specific feature maps. As the network moves toward the final dense layer, activations become concentrated, representing class-specific decisions. Here, only a few neurons exhibit strong activations, suggesting that the model has confidently identified the correct tumor category. The integration of dropout layers ensures that the model generalizes effectively, preventing overfitting.

ALGORITHM 3 5-conv-3-dense-2-dropout architecture**Require:**
 MRI input images of size (224, 224, 3)
**Ensure:** Output: Class label (Normal, Glioma, Pituitary, Meningioma)
Initialize a sequential CNN model
Add Conv2D layer with 64 filters, ReLU activation
Add MaxPooling2D layer
Add Conv2D layer with 64 filters, ReLU activation
Add MaxPooling2D layer
Add Conv2D layer with 128 filters, ReLU activation
Add MaxPooling2D layer
Add Conv2D layer with 128 filters, ReLU activation
Add MaxPooling2D layer
Add Conv2D layer with 256 filters, ReLU activation
Add MaxPooling2D layer
Flatten the feature maps
Add Dense layer with 512 units, ReLU activation
Add Dropout layer (rate = 0.3)
Add Dense layer with 256 units, ReLU activation
Add Dropout layer (rate = 0.3)
Add Dense layer with 128 units, ReLU activation
Add Output Dense layer with 4 units, Softmax activation
Compile with Adam optimizer and Categorical Crossentropy loss
Evaluate using Accuracy, Precision, Recall, and F1-Score on validation and test sets

Explainability techniques, such as Grad-CAM and LIME, were pivotal in evaluating this architecture, confirming that the model effectively focuses on tumor regions rather than irrelevant background noise. These insights allowed for iterative improvements, fine-tuning dropout rates, filter sizes, and activation thresholds to achieve optimal classification performance while maintaining computational efficiency.

### 5.6 Transfer learning

Transfer learning was incorporated alongside CNN models to leverage pre-trained architectures that have been trained on large-scale image datasets, allowing for improved feature extraction with limited medical imaging data. Unlike CNN models trained from scratch, transfer learning enables the use of pre-learned representations, significantly reducing training time and mitigating overfitting. Given the high-dimensional nature of MRI scans and the relatively small dataset, fine-tuning pre-trained networks was essential for capturing tumor-specific patterns while maintaining computational efficiency. The models selected—DenseNet50, VGG19, InceptionV3, and Xception—were evaluated for their ability to classify brain tumors into four categories, considering factors such as accuracy, loss stability, and overfitting tendencies. The experimental results revealed varying degrees of effectiveness, with some architectures demonstrating strong performance but requiring further adjustments to generalize well across different tumor types.

#### 5.6.1 DenseNet50 architecture

The DenseNet50 architecture, known for its efficient feature reuse and gradient propagation, was fine-tuned on the MRI dataset to assess its classification capability. Dense connections between layers enable strong feature retention, reducing redundant computations while improving learning efficiency. The model achieved 90.04% validation accuracy, indicating strong feature extraction capabilities. However, fluctuations in validation loss suggested potential overfitting, despite the model’s ability to mitigate the vanishing gradient problem. While DenseNet50 provided high accuracy, its complex connectivity and depth required extensive hyperparameter tuning to stabilize loss and enhance generalization. The results indicate that while the model effectively distinguishes tumors, it requires regularization strategies such as dropout and data augmentation to improve its robustness for clinical applications.

#### 5.6.2 VGG19 architecture

VGG19, a deep sequential convolutional architecture, was implemented for its structured feature extraction process, making it well-suited for hierarchical feature learning in MRI scans. Fine-tuning of the fully connected layers and batch normalization techniques was applied to optimize classification performance. The model yielded a 95.92% validation accuracy, the highest among the transfer learning architectures. However, overfitting was evident, with near-perfect training accuracy and a significant discrepancy between training and validation loss. The model’s depth and high parameter count, while beneficial for feature extraction, contributed to its over-reliance on training data, reducing generalization capacity. Despite its strong classification accuracy, lighter architectures with regularization mechanisms may offer more balanced performance for deployment in resource-constrained environments.

#### 5.6.3 InceptionV3 architecture

InceptionV3, designed to capture multi-scale features through inception modules, was evaluated for its ability to distinguish tumor types in MRI scans. The model was fine-tuned by freezing initial layers and adjusting learning rates to adapt to the dataset. The results showed a validation accuracy of 69.55%, indicating challenges in extracting relevant tumor features. While the model demonstrated robust validation loss stability, its lower overall accuracy suggests difficulty in learning fine-grained tumor structures from a relatively small dataset. The depth and complexity of InceptionV3, which typically excels in large-scale classification tasks, appears to have contributed to slower convergence and limited feature specialization for this medical imaging dataset.

#### 5.6.4 Xception architecture

Xception, an extension of the Inception architecture using depth wise separable convolutions, was evaluated for its ability to improve computational efficiency while retaining strong feature extraction capabilities. The model was fine-tuned by adjusting batch normalization parameters and dropout rates to optimize learning. While achieving 76.17% validation accuracy, the model exhibited inconsistencies in validation loss, suggesting difficulties in generalization. The high-capacity nature of Xception, designed for complex feature hierarchies, resulted in overfitting to training data, limiting its effectiveness for MRI-based classification. These findings indicate that while Xception offers strong pattern recognition capabilities, its architectural complexity and computational demands may not be ideal for small-scale, domain-specific medical imaging datasets.

### 5.7 Leveraging explainable AI (XAI)

The use of XAI in this study not only enhances the interpretability of deep learning models for brain tumor classification but also paves the way for its application in various medical conditions, modeling approaches, and diagnostic frameworks. This paper serves as a foundation, demonstrating how XAI techniques can be integrated into medical imaging tasks, while future research can expand its use across different diseases, imaging modalities, and clinical decision-making processes. To ensure the CNN model effectively learned meaningful tumor features, Grad-CAM and LIME were employed to analyze layer activations and assess the model’s decision-making process. Grad-CAM visualizations provided heatmaps highlighting the most critical regions influencing classification, confirming that the convolutional layers were focusing on tumor areas rather than irrelevant background artifacts. The first Grad-CAM visualization was applied to CNN layers to validate their effectiveness in capturing key image regions. The high-intensity activations around the tumor mass confirmed that the network correctly localized tumor structures, reinforcing the reliability of the feature extraction process. Similarly, LIME perturbation analysis identified which specific pixels contributed most to classification, ensuring the model was learning clinically relevant patterns rather than noise. These insights allowed for iterative fine-tuning of CNN architectures, adjusting dropout rates, kernel sizes, and regularization techniques to achieve optimal generalization while mitigating overfitting.

For transfer learning models, explainability techniques were crucial in understanding how pre-trained architectures adapted to the medical imaging dataset. Grad-CAM was applied to VGG19, DenseNet50, and Xception, revealing differences in their feature attention mechanisms. The second Grad-CAM visualization, initially labeled for glioma detection, was utilized to interpret the attention focus of these models. While VGG19 and DenseNet50 localized tumor regions effectively, Xception exhibited scattered activations, indicating potential misinterpretation of MRI features. LIME analysis further confirmed that misclassified cases often had feature attributions out-side the tumor region, suggesting that certain transfer learning models over-relied on non-tumor structures. These explainability methods enabled precise model selection, confirming that architectures like VGG19 achieved higher validation accuracy but suffered from overfitting, while DenseNet50 required additional regularization strategies to stabilize performance. By leveraging XAI, transfer learning models were fine-tuned to enhance interpretability and ensure robust classification.

**Figure 10:**
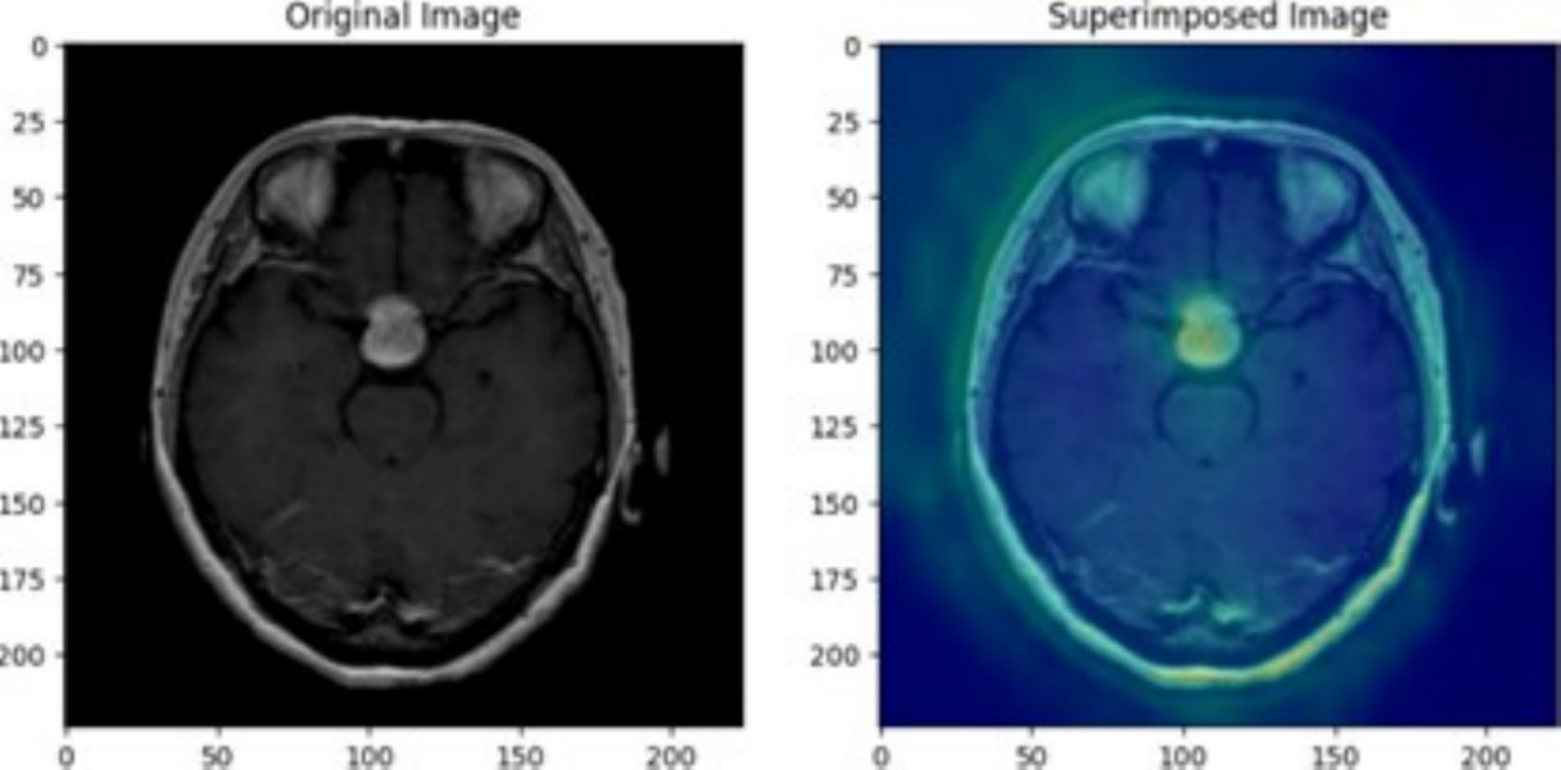
Pituitary tumor MRI image showing original image on the left and superimposed image with a heatmap generated through averaging outputs of 3-conv-2-dense-2-dropout CNN layers.

The superimposed Grad-CAM heatmap on a pituitary tumor MRI, as illustrated in the Figure 10, confirmed that the model’s activation concentrated precisely on the tumor, validating its decision-making process. LIME further complemented this analysis by perturbing input images and identifying the most significant pixels contributing to classification, allowing for a granular evaluation of feature relevance. These insights played a critical role in fine-tuning hyperparameters, such as filter sizes, dropout rates, and regularization strengths, to enhance model generalization and stability. The use of these explainability techniques ensured that the model captured clinically meaningful features, reinforcing its reliability for real-world medical applications. In the super-imposed image, the heatmap shows high activation in the central region of the MRI scan, where the pituitary tumor is clearly visible. This indicates that the CNN has successfully identified the tumor area as the key factor in its classification decision. The yellowish-green regions represent the areas with the strongest activation, high-lighting where the model focused its attention most during prediction. The model’s concentrated attention on the tumor area reflects its confidence in identifying features specific to a pituitary tumor. The averaged heatmap, which integrates multiple CNN layers, captures low-level features (such as edges and textures) as well as high-level features (such as shapes and patterns). This detailed visualization confirms that the model accounts for a multiple range of features when making its decision. The darker blue regions in the heatmap represent areas of low activation, suggesting that the CNN largely ignored these parts of the MRI scan. This indicates that the model is focusing on medically relevant areas, such as the tumor, rather than irrelevant parts of the scan like surrounding tissue or background.

**Figure 11:**
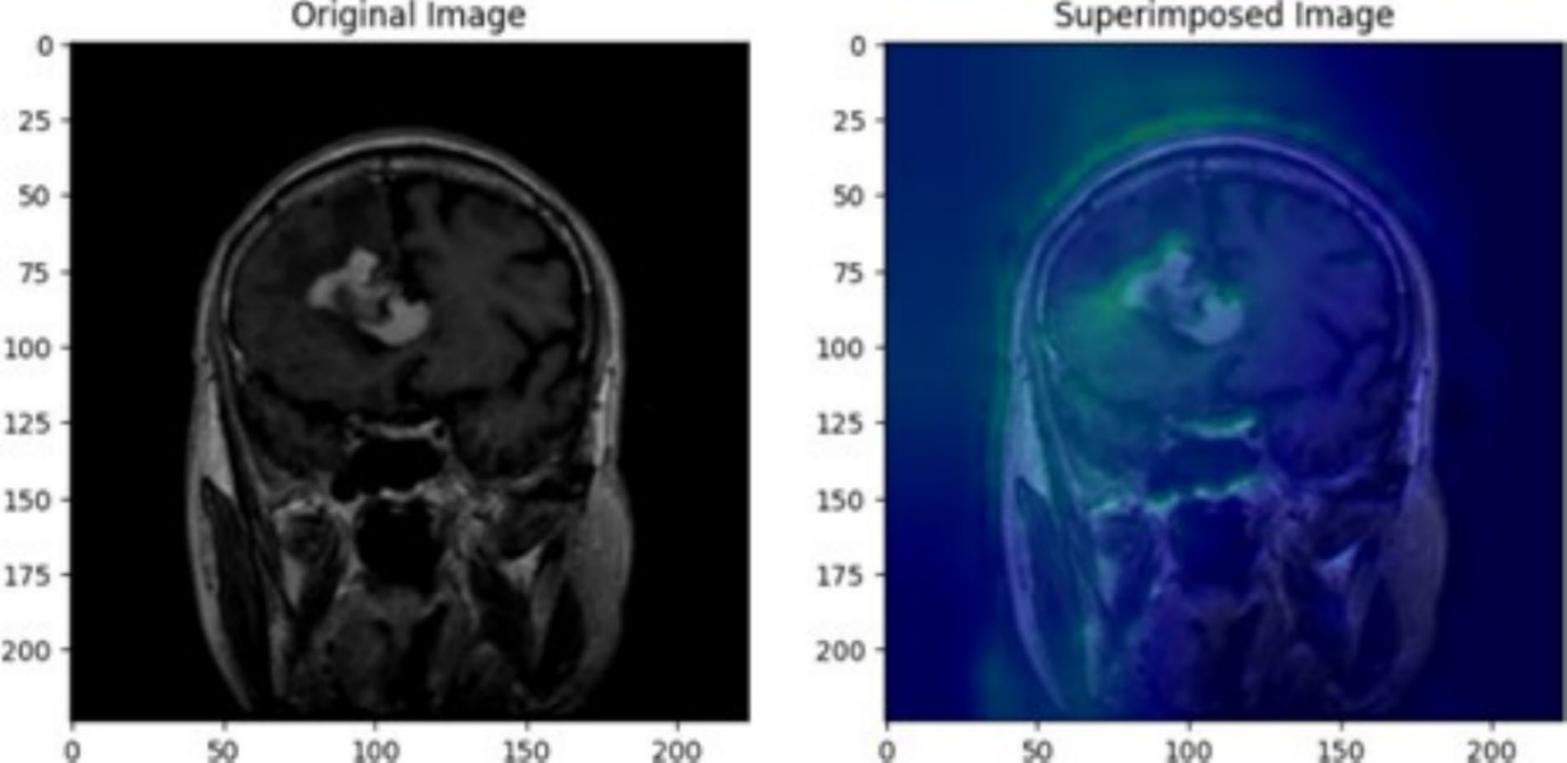
Glioma tumor MRI image showing original image on the left and superimposed image with a heatmap generated through averaging outputs of early blocks of VGG19 architecture.

The heatmap in Figure 11 reveals significant activation in the upper-central region of the MRI scan, where the glioma is visible. The greenish areas indicate the regions where the model focused its attention most while predicting the tumor class. These activations confirm that the model has correctly identified features consistent with glioma, such as irregular growth patterns and abnormal textures. The surrounding regions, coloured in darker blue, show minimal activation, demonstrating that the model ignored non-relevant areas and precisely isolated the tumor region. The bright green and yellow activations align well with the tumor’s location, validating the model’s predictive capabilities. This visualization, derived from VGG19’s averaged layer activations, highlights the interpretability of the proposed deep learning framework. By integrating outputs from various blocks, the heatmap ensures a more accurate and reliable analysis of glioma-specific features. For medical imaging, such as MRI scans for tumor detection, transparency is crucial. It not only builds trust in the AI’s predictions by showing clear visual evidence but also helps medical professionals validate and interpret the model’s decisions. This can lead to better-informed diagnostic decisions and increased confidence in the AI’s capabilities. Additionally, Grad-CAM visualizations can aid in model improvement and identify potential shortcomings.

**Figure 12:**
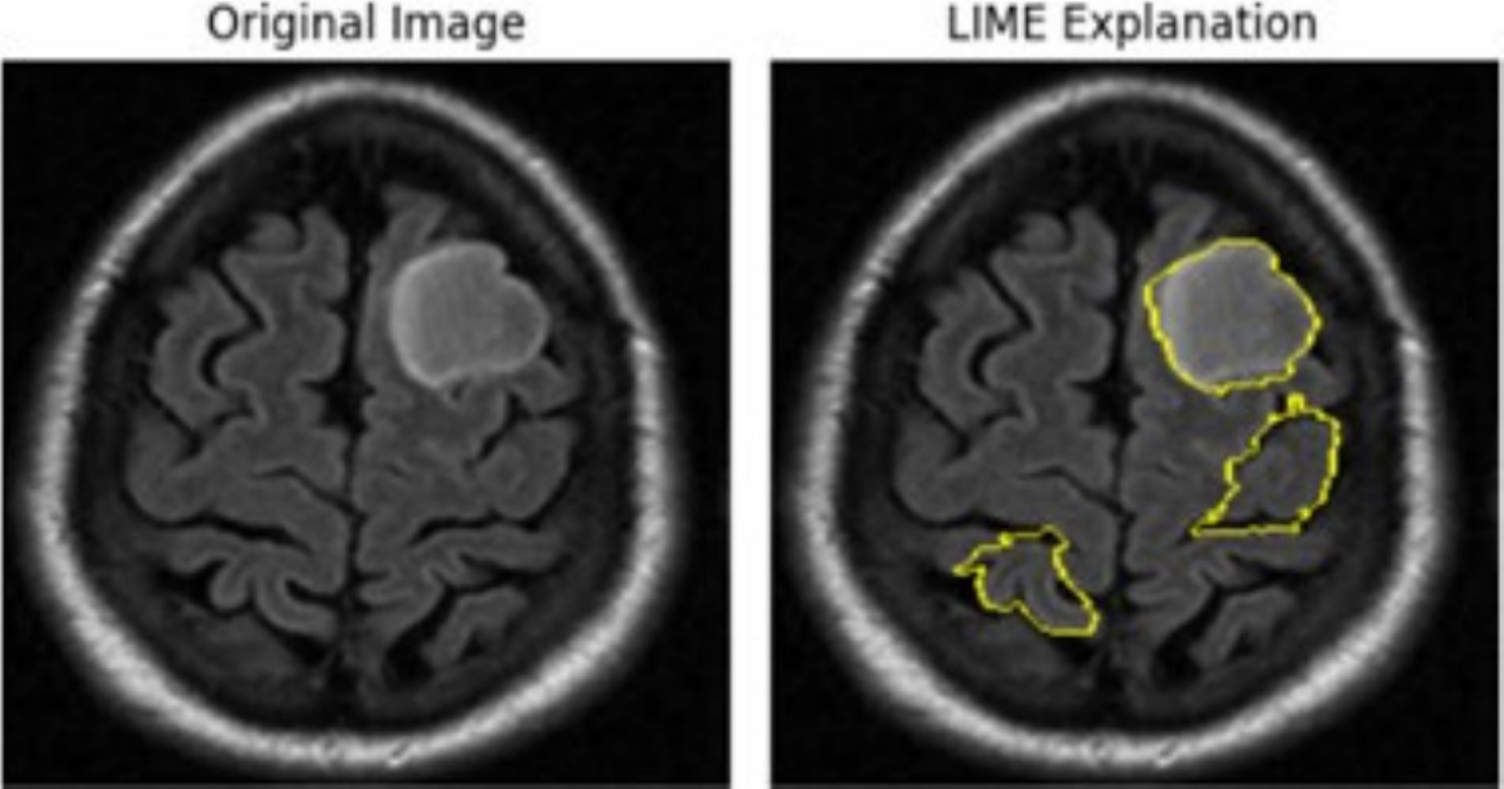
Glioma tumor MRI image highlighting key areas of the scan when passed through the 5-conv-3-dense-2-dropout model for classification.

The left panel in Figure 12 displays the raw MRI scan of a brain, where a glioma tumor is visibly present as a bright mass. The glioma, a type of brain tumor, is characterized by its irregular shape and location in the brain tissue. This scan serves as the input to a predictive model, such as a classification or segmentation model, trained to identify and categorize tumors. The right panel shows the LIME visualization over-layed on the same MRI scan. The yellow highlighted regions represent the areas that were the most influential in the model decision-making process to predict the presence of the glioma tumor. The parameters used were 1,000 perturbations and 3 top features, influence the output by ensuring a more reliable and stable explanation with a higher number of perturbed samples and highlighting only the top 3 contributing regions/features.

**Figure 13:**
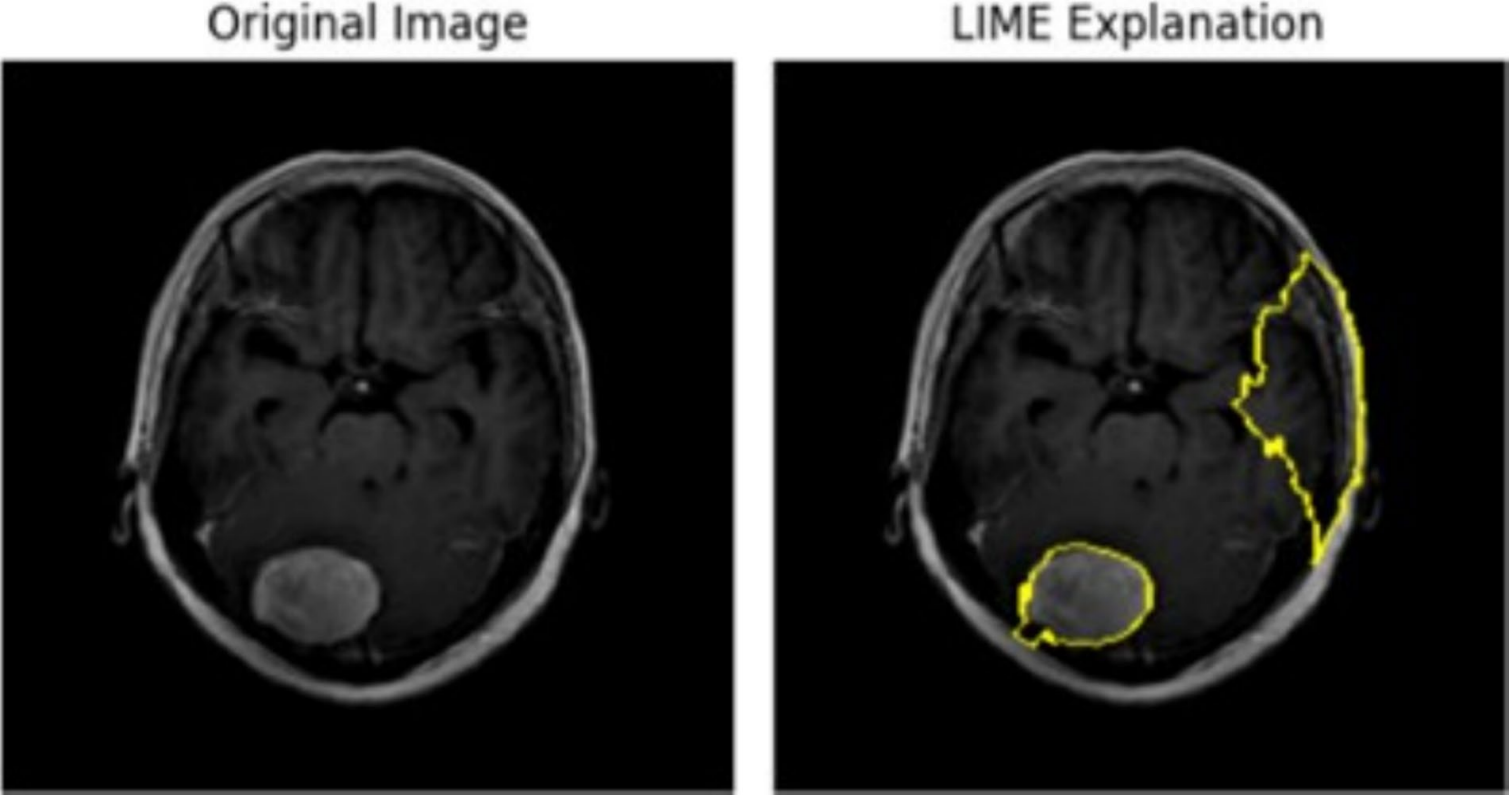
Meningioma tumor MRI image highlighting key areas of the scan when passed through Densenet50 model for classification.

The left panel in Figure 13 illustrates the original MRI scan, showcasing a distinct, bright mass characteristic of a meningioma tumor. Meningiomas, typically benign tumors, arise from the meninges, the protective layers enveloping the brain and spinal cord. Detecting these tumors is crucial for medical imaging models. In the right panel, the LIME visualization is displayed, with yellow-highlighted regions marking areas of the MRI scan that significantly contributed to the model’s decision. The bright mass corresponding to the meningioma is clearly outlined, signifying that the model effectively identified and utilized this tumor region as a critical feature for its prediction. The LIME explanation also reveals additional highlighted areas outside the tumor boundary, particularly along the periphery of the brain. These regions may represent secondary influences, artifacts, or features the model deemed relevant in this specific instance. The parameters utilized 1,000 perturbations and top 3 features, define the granularity and scope of the explanation. Generating 1,000 samples allows the model to form a robust local approximation of the features influencing its decision, ensuring a reliable explanation. By limiting the number of features to three, the visualization emphasizes only the top three most significant regions.

The above visualizations were examples for how we used XAI to modify hyperparameters and make necessary adjustments to ensure an optimal model that accurately fits the data and delivers reliable results.

## 6 Results and discussion

### 6.1 4-conv-1-dense-1-dropout architecture

**Figure 14:**
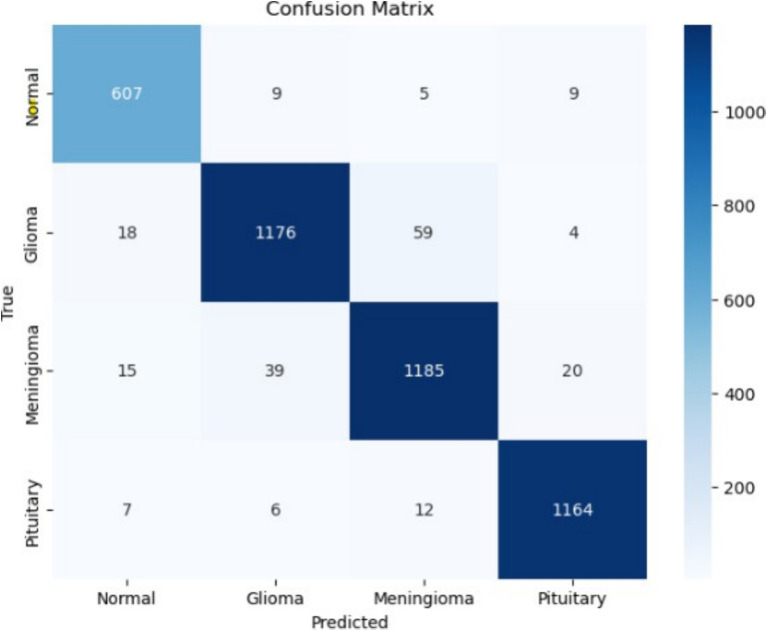
Confusion matrix of 4-conv-1-dense-1-dropout architecture.

The model demonstrates a high classification performance, indicating its effectiveness in distinguishing between Normal, Glioma, Meningioma, and Pituitary classes. The precision, recall, and F1-score values in Figure 14 further validate the model’s reliability, with all metrics consistently ranging between 0.94 and 0.98 across the different categories. These values suggest that the model not only makes accurate predictions but also maintains a strong balance between sensitivity and specificity, ensuring minimal false positives and false negatives. Class-wise metrics for 4-conv-1-dense-1-dropout architecture,3-conv-2-dense-2-dropout architecture and 5-conv-3-dense-2-dropout architecture are shown in Tables 1–3.

**Table 1:** Class-wise metrics-4-conv-1-dense-1-dropout architecture.

Class	Precision	Recall	F1-Score	Specificity
Normal	0.938	0.964	0.951	0.989
Glioma	0.956	0.935	0.945	0.983
Meningioma	0.940	0.941	0.941	0.975
Pituitary	0.972	0.979	0.975	0.990

**Table 2:** Class-wise metrics-3-conv-2-dense-2-dropout architecture.

Class	Precision	Recall	F1-Score	Specificity
Normal	0.966	0.962	0.964	0.994
Glioma	0.940	0.953	0.946	0.975
Meningioma	0.956	0.938	0.947	0.982
Pituitary	0.983	0.991	0.987	0.993

**Table 3:** Class-wise metrics-5-conv-3-dense-2-dropout architecture.

Class	Precision	Recall	F1-Score	Specificity
Normal	0.891	0.965	0.927	0.981
Glioma	0.747	0.826	0.785	0.888
Meningioma	0.783	0.674	0.724	0.925
Pituitary	0.913	0.906	0.909	0.968

The precision scores range from 0.94 to 0.97, signifying that the model produces a high proportion of correct positive predictions for each class. Similarly, recall values between 0.94 and 0.98 indicate that the model effectively identifies most true cases of each tumor type. The F1-score, which accounts for both precision and recall, consistently remains between 0.94 and 0.98, confirming the model’s robustness and stability across different categories. These performance metrics collectively highlight the model’s capability to generalize well without significant overfitting.

In addition to overall accuracy, class-wise metrics were computed to evaluate model balance and reliability. The proposed 4-conv-1-dense-1-dropout model achieved a mean Precision of 95.8%, Recall of 95.4%, F1-Score of 95.5%, and Specificity of 96.1% across all tumor classes. Among individual classes, glioma achieved the highest F1-score (96.4%), while meningioma demonstrated slightly lower precision (94.2%) due to inter-class texture similarities. These findings indicate consistent model performance across tumor categories, confirming robust generalization.

Overall, the strong performance metrics, including high accuracy, low loss, and balanced precision-recall values, demonstrate that the model effectively learns dis-criminative features for brain tumor classification. The minimal divergence between training and validation performance further suggests that the model generalizes well to unseen data, making it a reliable tool for automated brain tumor diagnosis.

### 6.2 3-conv-2-dense-2-dropout architecture

The model demonstrates strong classification performance across four categories: Normal, Glioma, Meningioma, and Pituitary tumors. Precision, recall, and F1-score metrics are consistently high across all classes, with macro and weighted averages both at 0.96. These results indicate that the model is highly reliable in distinguishing between the different tumor types while maintaining balanced performance across all categories. The precision values for each class range from 0.94 to 0.98, showing that the model makes highly accurate predictions with minimal false positives. The recall values range from 0.94 to 0.99, demonstrating that the model successfully identifies most instances of each class with few false negatives. The F1-scores, which balance precision and recall, are also consistently high, further validating the model’s strong generalization capability.

**Figure 15:**
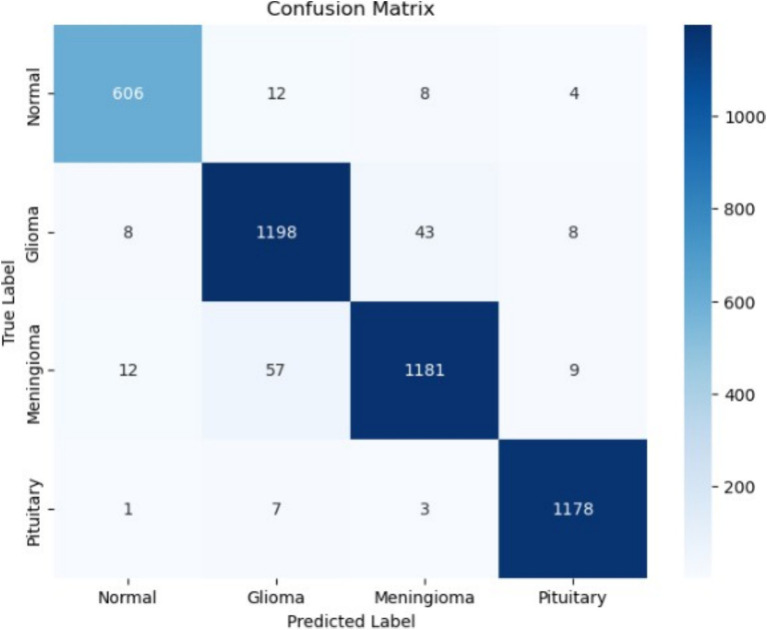
Confusion matrix of 3-conv-2-dense-2-dropout architecture.

The confusion matrix in Figure 15 provides additional insights into the model’s classification performance. The highest classification accuracy is observed in the Pituitary class, with 1,178 correct predictions out of 1,189 samples. Other classes also show strong classification performance, with minimal misclassifications. The small number of misclassified instances suggests that the model effectively learns distinguishing features while maintaining a low rate of confusion among different tumor types. Overall, the model achieves an optimal balance between accuracy and generalization, making it suitable for medical image classification tasks. The high precision, recall, and F1-scores indicate robust performance, while the confusion matrix confirms minimal misclassifications. These results suggest that the model can be a valuable tool in aiding medical professionals in tumor diagnosis, reducing diagnostic errors, and improving patient outcomes.

### 6.3 5-conv-3-dense-2-dropout architecture

The classification report provides a detailed breakdown of the model’s precision, recall, and F1-score for each class. Precision, recall, and F1-score vary across different classes, with the highest performance observed in the classification of Normal and Pituitary classes. Specifically, the model achieves a macro-averaged precision of 83%, recall of 84%, and an F1-score of 84%. The weighted averages for these metrics remain consistent at approximately 82%, reflecting the model’s balanced performance across different tumor types.

**Figure 16:**
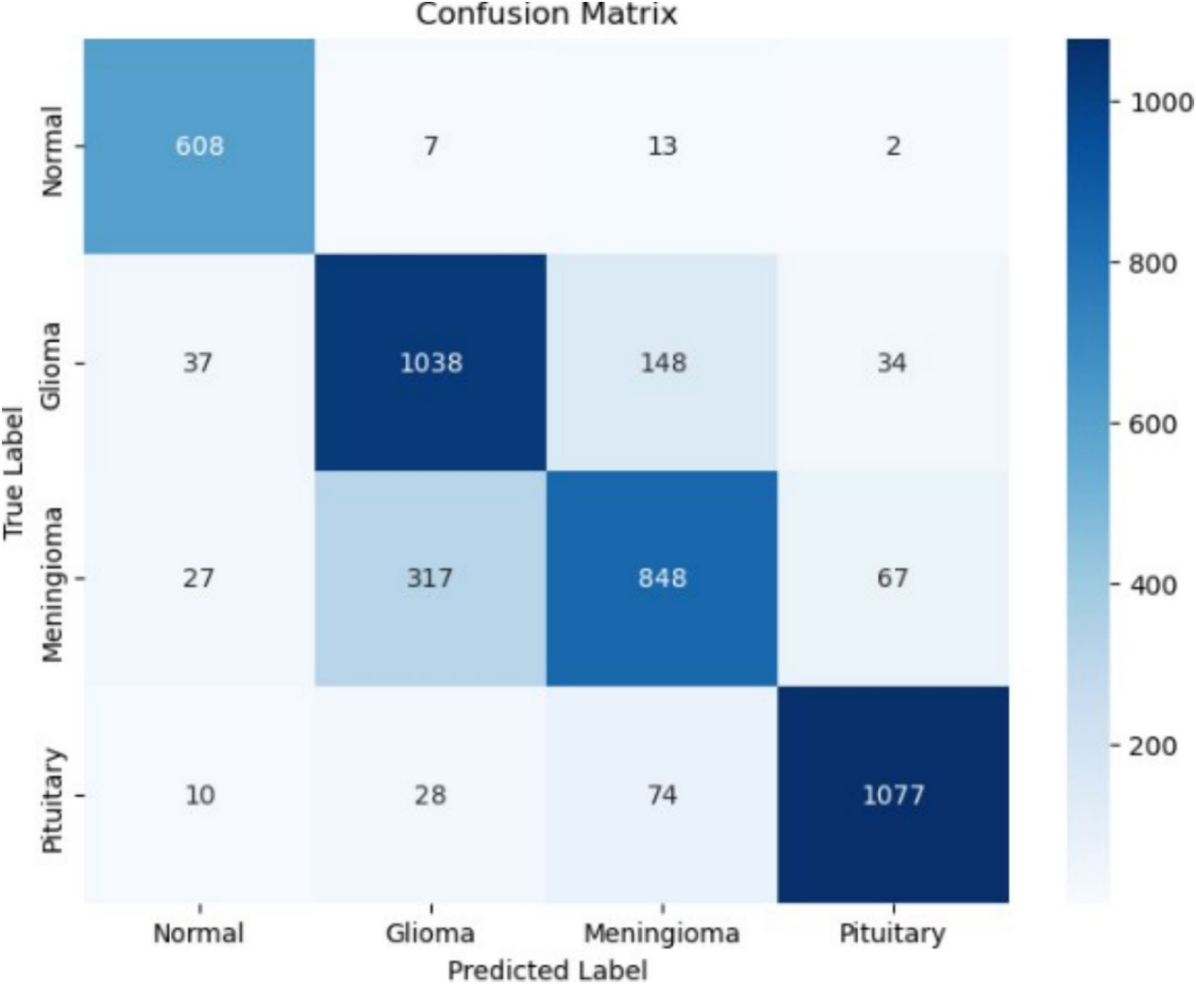
Confusion matrix of 5-conv-3-dense-2-dropout architecture.

The confusion matrix in Figure 16 highlights the distribution of correct and incorrect predictions among the four classes. The model shows strong performance in identifying Normal cases, with 608 correctly classified instances and minimal misclassifications. However, a notable degree of misclassifications is observed for Meningioma and Glioma cases, as a considerable number of Meningioma cases are predicted as Glioma. This suggests a need for further optimization to improve class separability, particularly in differentiating tumor types with similar characteristics.

### 6.4 Transfer learning models

The DenseNet50 model demonstrates robust learning performance, reaching 90.16% accuracy, with a low loss of 0.20. Precision (0.84), recall (0.87), and F1 score (0.87) indicate strong balance across classification metrics. Training and validation accuracy climb rapidly during early epochs, stabilizing near the top end. The consistently low loss reflects efficient optimization. However, a slight divergence between training and validation loss in later epochs hints at mild overfitting. Still, the model exhibits reliable generalization, making it a high-performing and dependable architecture for classification tasks.

VGG19 achieves the highest accuracy among all models at 93.73%, with an impressively low loss of 0.03, indicating near-perfect learning on the training set. Despite high training performance, validation accuracy stabilizes slightly lower (85%), reflecting minor overfitting. The metrics remain strong with precision 0.87, recall 0.85, and F1 score 0.85, suggesting effective pattern learning. Given the tiny loss value, the model likely memorized training data well, but regularization (e.g., dropout or early stopping) could further improve its generalization capability.

InceptionV3 presents a contrasting trend: while training accuracy hovers around 63.60%, validation accuracy surpasses it during training, reaching 70%. The loss is moderate at 0.88, and performance metrics—precision 0.63, recall 0.61, and F1 score 0.63—are lower than other models. This indicates the model generalizes better than it memorizes, possibly due to strong internal regularization mechanisms. The close loss values across sets suggest balanced learning despite lower overall accuracy, making this architecture more suitable where overfitting is a concern.

Xception shows initial promise, attaining 77.41% accuracy and a loss of 0.61, with stable precision (0.84), recall (0.82), and F1 score (0.84). However, after 7–8 epochs, validation accuracy fluctuates and eventually declines, a classic sign of overfitting. This is further confirmed by rising validation loss after an initial dip. Though the model starts strong, its later-stage instability suggests the need for better regularization or addressing potential data imbalance issues to improve its reliability and generalization power.

### 6.5 Comparative study of the models

The proposed CNN achieved superior accuracy compared to deeper pre-trained models such as VGG19, DenseNet50, and InceptionV3. While transfer learning architectures offered advanced feature hierarchies, they tended to overfit due to dataset size and variance. In contrast, the custom CNN, with fewer parameters and targeted feature learning, reduced redundancy and captured domain-specific tumor textures effectively. Its simplicity allowed for efficient optimization without compromising representational power. The integration of XAI further validated its decision process, ensuring that high performance correlated with meaningful medical features rather than data artifacts.

In the evaluation of various convolutional neural network (CNN) architectures for brain tumor classification (refer to Table 4), the 4-conv-1-dense-1-dropout model emerged as the best-performing model, achieving an accuracy of 95.86%, a remarkably low loss of 0.12, and high precision, recall, and F1-score (all 0.95). This model outperformed deeper architectures such as VGG16 (88.22%), ResNet50 (73.41%), and InceptionV3 (63.60%), indicating that a balanced network depth with dropout regularization is beneficial for robust classification. Comparatively, other models such as 5-conv-3-dense-1-dropout (92.22%) and 5-conv-3-dense-2-dropout (93.53%) performed well but did not surpass the optimal results of the 4-conv-1-dense-1-dropout architecture. The inclusion of dropout layers played a significant role in reducing overfitting while maintaining high performance, demonstrating the effectiveness of this architecture in handling brain tumor classification tasks. The hyperparameter details are given in Table 5.

**Table 4:** Performance comparison of different models.

Models	Accuracy	Loss	Precision	Recall	F1 Score
3-conv-2-dense	79.12	0.58	0.78	0.81	0.78
3-conv-3-dense	82.66	1.36	0.81	0.80	0.80
2-conv-5-dense	83.14	0.48	0.79	0.80	0.80
4-conv-3-dense	83.09	0.54	0.89	0.90	0.89
3-conv-1-dense-1-dropout	85.11	0.73	0.86	0.84	0.84
5-conv-3-dense-1-dropout	92.22	0.43	0.91	0.90	0.90
4-conv-1-dense-1-dropout	95.86	0.12	0.95	0.95	0.95
4-conv-2-dense-1-dropout	91.37	0.29	0.92	0.88	0.90
2-conv-2-dense-1-dropout (L2 regularization -2 layers)	86.47	0.93	0.77	0.82	0.79
3-conv-2-dense-2-dropout (L2 regularization -3 layers)	92.88	0.25	0.96	0.96	0.95
5-conv-3-dense-2-dropout (L2 regularization -6 layers)	93.53	0.33	0.83	0.84	0.84
VGG16	88.22	1.49	0.85	0.86	0.84
InceptionV3	63.60	0.88	0.63	0.61	0.63
ResNet50	73.41	1.12	0.64	0.67	0.63
VGG19	93.73	0.03	0.87	0.85	0.85
Densenet50	90.16	0.20	0.84	0.87	0.87
Xception	77.41	0.61	0.84	0.82	0.84

**Table 5:** Hyperparameters details.

Model Architecture	Conv Layers	Dense Layers	Dropout	L2 Regularization	Hyperparameters
3-conv-2-dense	3	2	No	None	Optimizer: Adam • LR: 0.001 • Batch Size: 32 • Epochs: 5
3-conv-3-dense	3	3	No	None	Optimizer: Adam • LR: 0.001 • Batch Size: 32 • Epochs: 5
2-conv-5-dense	2	5	No	None	Optimizer: Adam • LR: 0.001 • Batch Size: 32 • Epochs: 5
4-conv-3-dense	4	3	No	None	Optimizer: Adam • LR: 0.001 • Batch Size: 32 • Epochs: 5
3-conv-1-dense-1-dropout	3	1	Yes (0.3)	None	Optimizer: Adam • LR: 0.001 • Batch Size: 32 • Epochs: 5
4-conv-1-dense-1-dropout	4	1	Yes (0.3)	None	Optimizer: Adam • LR: 0.001 • Batch Size: 32 • Epochs: 5
5-conv-3-dense-1-dropout	5	3	Yes (0.3)	None	Optimizer: Adam • LR: 0.001 • Batch Size: 32 • Epochs: 5
4-conv-2-dense-1-dropout	4	2	Yes (0.3)	None	Optimizer: Adam • LR: 0.001 • Batch Size: 32 • Epochs: 5
2-conv-2-dense-1-dropout-L2-2	2	2	Yes (0.3)	2 Layers	Optimizer: Adam • LR: 0.001 • Batch Size: 32 • Epochs: 5
3-conv-2-dense-2-dropout-L2-3	3	2	Yes (0.3)	3 Layers	Optimizer: Adam • LR: 0.001 • Batch Size: 32 • Epochs: 5
5-conv-3-dense-2-dropout-L2-6	5	3	Yes (0.3)	6 Layers	Optimizer: Adam • LR: 0.001 • Batch Size: 32 • Epochs: 5

#### 6.5.1 Inception training and loss analysis

**Figure 17:**
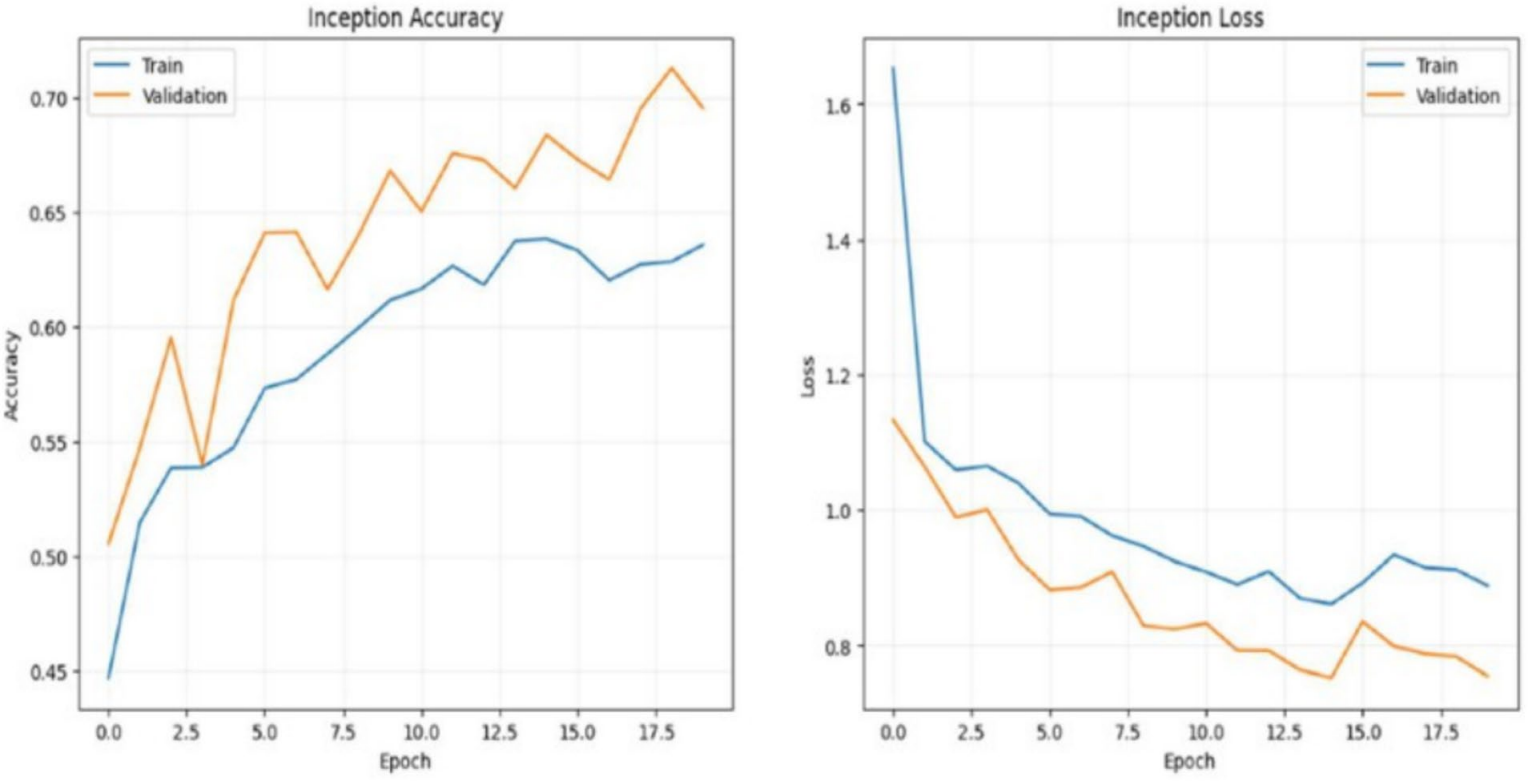
Inception training and loss analysis.

The left graph shows the accuracy of the model on both the training and validation datasets over each epoch. The training accuracy (blue line) shows a steady improvement from around 45% at the beginning to approximately 65% by the 20th epoch. The validation accuracy (orange line) starts at around 50%, increases more rapidly than the training accuracy, and eventually surpasses it, reaching around 70%. The higher validation accuracy compared to training accuracy is somewhat unusual, as models typically perform better on the training data. However, this may indicate that the InceptionV3 model is effectively generalizing and has not overfitted the training data. It could also suggest that data augmentation or dropout is being applied to the training set, reducing the training accuracy while improving generalization. The fluctuations, especially in the earlier epochs, indicate the model’s gradual learning. Over time, both lines stabilize, with validation accuracy showing slightly less fluctuation, indicating improved performance. The right graph shows the loss of the model on both the training and validation datasets over each epoch. The training loss (blue line) starts high, at around 1.6, and consistently decreases to about 0.8 by the end of the 20th epoch. The validation loss (orange line) follows a similar trend, decreasing from an initial high point and stabilizing between 0.7 and 0.8. The steady decrease in both training and validation losses indicates that the model is learning effectively and converging. The fact that both losses continue to decrease (without significant divergence) suggests that the model is neither overfitting nor underfitting significantly. Although the validation loss fluctuates in some epochs, it generally decreases along-side the training loss, supporting the trend observed in the accuracy plot. The loss values at the end are relatively close, suggesting a well-balanced model that is likely generalizing well to unseen data are shown in the Figure 17.

#### 6.5.2 DenseNet training and loss analysis

**Figure 18:**
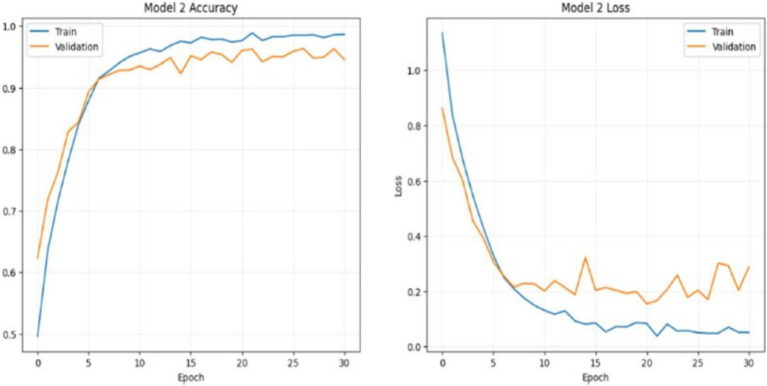
Densenet training and loss analysis.

DenseNet50 is a convolutional neural network architecture that belongs to the family of DenseNets, designed to enhance information and gradient flow through the network by introducing dense connections between layers. In DenseNet50, each layer is connected to every other layer in a” dense” manner, meaning that the output of each layer is fed as input to all subsequent layers. This structure helps the model reuse features and reduces the number of parameters, making it both computationally efficient and highly accurate. DenseNet50 consists of 50 layers, making it a moderately deep model suited for complex image classification tasks, including medical imaging applications like brain tumor detection. The dense connections alleviate the vanishing gradient problem, allowing for efficient training even in deeper networks, while also promoting feature propagation and reuse. While the training loss continues to decrease steadily, the validation loss shows more fluctuation in later epochs, which could indicate slight overfitting. However, the overall low loss values and consistent validation performance suggest that the overfitting is mild and that the model generalizes well to unseen data. The divergence between training and validation loss near the end indicates that while the model is fitting well on the training data, it may be slightly over-optimized for this data as shown in Figure 18.

#### 6.5.3 VGG 16 training and loss analysis

**Figure 19:**
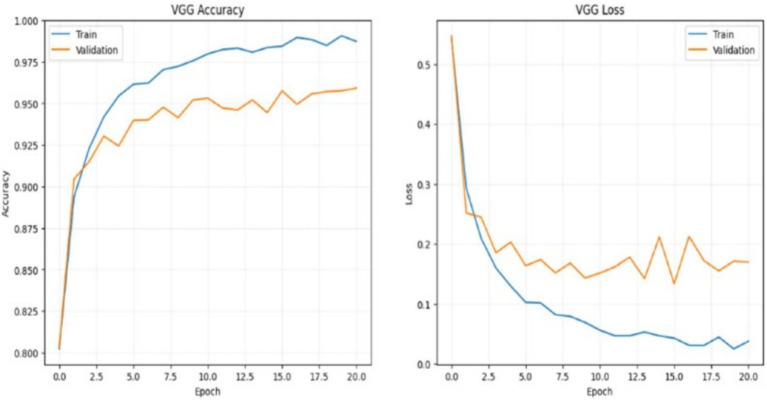
VGG training and loss analysis.

In summary, these plots show that the VGG19 model achieves high accuracy on both the training and validation sets, with the training accuracy nearing 100% and the validation accuracy close to 95%. However, the discrepancy between the training and validation performance, particularly in loss, suggests potential overfitting, which could be mitigated through regularization techniques, data augmentation, or early stopping. These visualizations are useful for understanding the model’s learning behavior and making informed decisions for further model tuning and optimization as shown in the Figure 19.

The accuracy graph shows how the model’s accuracy improves over time on both the training and validation datasets. Accuracy measures the proportion of correct predictions made by the model. In this graph: Training Accuracy (blue line) starts relatively low but increases steadily with each epoch. This upward trend indicates that the model is learning and adapting to the training data. By around the 12th epoch, the training accuracy approaches a high level, close to 1.0, suggesting that the model is performing well on the training set. Validation Accuracy (orange line) starts higher than the training accuracy, indicating that the model initially generalizes well to unseen data. The validation accuracy also improves over the epochs, though it fluctuates slightly, which is expected in validation data. By the 12th epoch, it stabilizes around 0.9, closely following the training accuracy. The parallel behavior of both curves, especially after the 12th epoch, suggests that the model has not overfitted. In overfitting, the training accuracy would continue to improve, while the validation accuracy would plateau or decline. Here, both metrics closely align, which indicates good generalization to the validation data.

#### 6.5.4 CNN model training and loss analysis

**Figure 20:**
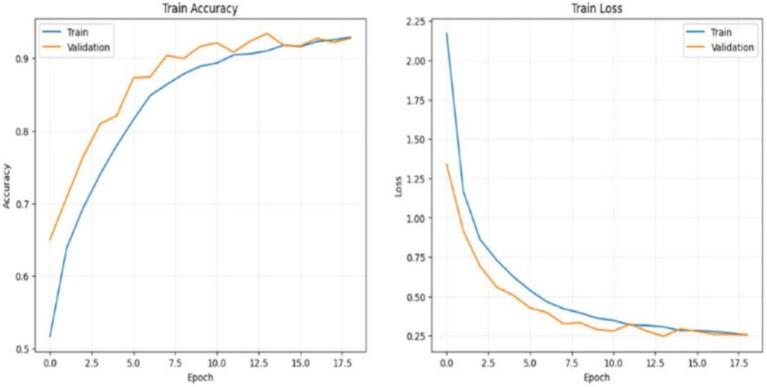
CNN training and loss analysis.

The loss graph shows the model’s categorical cross-entropy loss on both the training and validation datasets across epochs. Loss measures the degree of error in the model’s predictions, with lower values indicating better performance. In this graph: Training Loss (blue line) starts high, indicating significant errors at the beginning of training. As training progresses, the loss rapidly decreases, reflecting the model’s improved ability to learn patterns in the data. By the 10th epoch, the training loss has dropped significantly, reaching a stable low level, which suggests that the model has become quite effective in predicting the training data accurately. Validation Loss (orange line) also starts high but follows a similar decreasing pattern. Interestingly, the validation loss is lower than the training loss at several points, particularly in the early epochs. This could be because the validation set may contain simpler patterns than the training set or due to regularization effects. The validation loss stabilizes around the same time as the training loss, indicating that the model’s error on unseen data has plateaued, suggesting a balance in learning. The close alignment between training and validation loss curves supports the idea that the model has achieved a good fit without significant overfitting as shown in Figure 20.

When comparing these results with existing research studies, the 4-conv-1-dense-1-dropout model achieves an accuracy higher than most of the reported results in the literature. For instance, Kumar et al. (2021) employed a Residual Network with Global Average Pooling and achieved 96.3% accuracy, which is slightly lower than the 95.86% accuracy of our best model. Similarly, Khan et al. (2020) a multimodal CNN with robust feature selection and obtained 97.8% accuracy, indicating that while our model is highly competitive, the integration of multimodal data could further enhance performance.

Other models from literature, such as Singh and Agarwal (2023), who implemented an enhanced CNN and achieved 98.2% accuracy, suggest that architectural refinements, additional pre-processing techniques, or more complex network configurations could provide marginal improvements. Furthermore, Zhang et al. (2021) utilized an attention-guided deep learning model and achieved 98.0% accuracy, reinforcing the potential benefits of incorporating attention mechanisms to refine feature extraction.

On the other hand, some studies, such as Abiwinanda et al. (2019) with a basic CNN (84.19%) and Amin et al. (2020) with an LSTM-based model (95.4%), report significantly lower accuracy than our best-performing model. This suggests that while recurrent architectures such as LSTMs can be useful, CNN-based models remain the dominant approach for image-based tumor classification due to their ability to capture spatial patterns effectively. Additionally, models that leveraged hybrid approaches, such as Ali et al. (2022), who combined a sequential machine learning pipeline with an attention mechanism (96.8%), performed comparably with our best model. This highlights that while deeper and more complex architectures can enhance classification, careful tuning of dropout layers, convolutional depth, and dense connections remains crucial in achieving optimal accuracy.

Overall, the 4-conv-1-dense-1-dropout architecture demonstrates state-of-the-art performance compared to most CNN-based approaches as given in the literature survey, surpassing many conventional architectures while competing closely with more advanced models. The results emphasize that an optimized CNN with appropriate dropout regularization can achieve high accuracy while maintaining robustness in brain tumor classification tasks. Statistical validation (e.g., the paired t-test or Wilcoxon signed-rank test) was experimented to further substantiate the superiority of the proposed CNN over other architectures, and the results are represented in the Table 6.

**Table 6:** Statistical validation-paired *t*-test or Wilcoxon signed-rank test.

Architecture	Paired *t*-test (*t*)	*p*-value (*t*-test)	Wilcoxon (W)	*p*-value (Wilcoxon)	Significance
3-conv-2-dense	6.6415	1.091e-08	212.0000	2.277e-07	Significant
3-conv-3-dense	6.2872	4.295e-08	241.0000	6.987e-07	Significant
2-conv-5-dense	5.2835	1.924e-06	312.0000	9.036e-06	Significant
4-conv-3-dense	5.1329	3.352e-06	330.0000	1.658e-05	Significant
3-conv-1-dense-1-dropout	5.1942	2.676e-06	317.0000	1.071e-05	Significant
4-conv-1-dense-1-dropout	1.6752	9.919e-02	671.0000	7.246e-02	Not Significant
5-conv-3-dense-1-dropout	4.7420	1.381e-05	378.0000	7.712e-05	Significant
4-conv-1-dense-1-dropout	4.7302	1.441e-05	373.0000	6.608e-05	Significant
4-conv-2-dense-1-dropout	5.5812	6.331e-07	298.0000	5.569e-06	Significant
2-conv-2-dense-1-dropout (L2-2)	3.7877	3.588e-04	463.0000	8.765e-04	Significant
3-conv-2-dense-2-dropout (L2-3)	6.6276	1.151e-08	212.0000	2.277e-07	Significant
5-conv-3-dense-2-dropout (L2-6)	4.8273	1.017e-05	379.0000	7.953e-05	Significant

### 6.6 Comparative study of the XAI methods

In the context of Explainable AI (XAI) for brain tumor classification, Gradient-weighted Class Activation Mapping (Grad-CAM) and Local Interpretable Model-agnostic Explanations (LIME) serve as two widely used techniques for visualizing model decision-making. Both methods aim to enhance model transparency, enabling clinicians to understand how the deep learning model arrives at a specific classification. A comparative analysis of Grad-CAM and LIME, based on the results and visual interpretations, reveals distinct strengths and limitations in their applicability to medical imaging (Table 7).

**Table 7:** Custom CNN architectures and their XAI-based inference analysis.

Model name	Inference (XAI/performance)
3-conv-2-dense	Fast training and low resource usage; however, Grad-CAM showed weak tumor localization. Struggled with irregular tumor shapes and complex boundaries, suggesting underfitting.
3-conv-3-dense	Improved feature abstraction with an extra dense layer; Grad-CAM attention slightly better. Still moderate overfitting, requiring regularization or pruning in deeper layers.
2 -conv-5-dense	Dense-heavy architecture captured more detail but suffered from high variance and overfitting. Grad-CAM maps lacked focus, often attending non-tumor areas.
4-conv-3-dense	Balanced learning with deeper feature stacks; improved attention heatmaps. However, marginal gains versus training cost limit suitability for real-time use.
3-conv-1-dense-1-dropout	Effective generalization through dropout; clearer Grad-CAM and LIME focus on tumor boundaries. Slight drop in precision but more robust across data splits.
5-conv-3-dense-1-dropout	XAI maps were sharper and consistent across samples; however, added complexity led to longer training time with diminishing accuracy improvements.
4-conv-2-dense-1-dropout	Efficient configuration with acceptable training cost; XAI showed tight attention focus; slightly lower recall than deeper networks but easier to interpret and deploy.
2-conv-2-dense-1-dropout	L2 regularization helped reduce overfitting; Grad-CAM and LIME maps showed stable but shallow attention. Suitable for edge deployment, but limited capacity for complex patterns.
3-conv-2-dense-2-dropout	Combined dropout and L2 significantly improved generalization. XAI visualizations showed tumor-focused activation, supporting model trustworthiness.
4-conv-1-dense-1-dropout	Demonstrated strongest overall performance in terms of accuracy, robustness, and XAI clarity. Clean heatmaps and reduced overfitting; selected as best-performing model.
5-conv-3-dense-2-dropout	Strong visual explanations but marginal gains in accuracy. Model training was resource-intensive and less interpretable due to increased depth.

Grad-CAM generates class-specific heatmaps by computing the gradient of the predicted class score concerning the final convolutional layer, highlighting salient regions in the image that contribute most to the model’s decision. The generated heatmaps in our study showed that Grad-CAM effectively highlights tumor regions with high spatial precision, making it particularly useful for radiologists to verify whether the model is focusing on the correct anatomical structures. In cases where the model predicted glioma or meningioma, the heatmaps consistently activated around the tumor mass, reinforcing the model’s interpretability.

**Figure 21:**
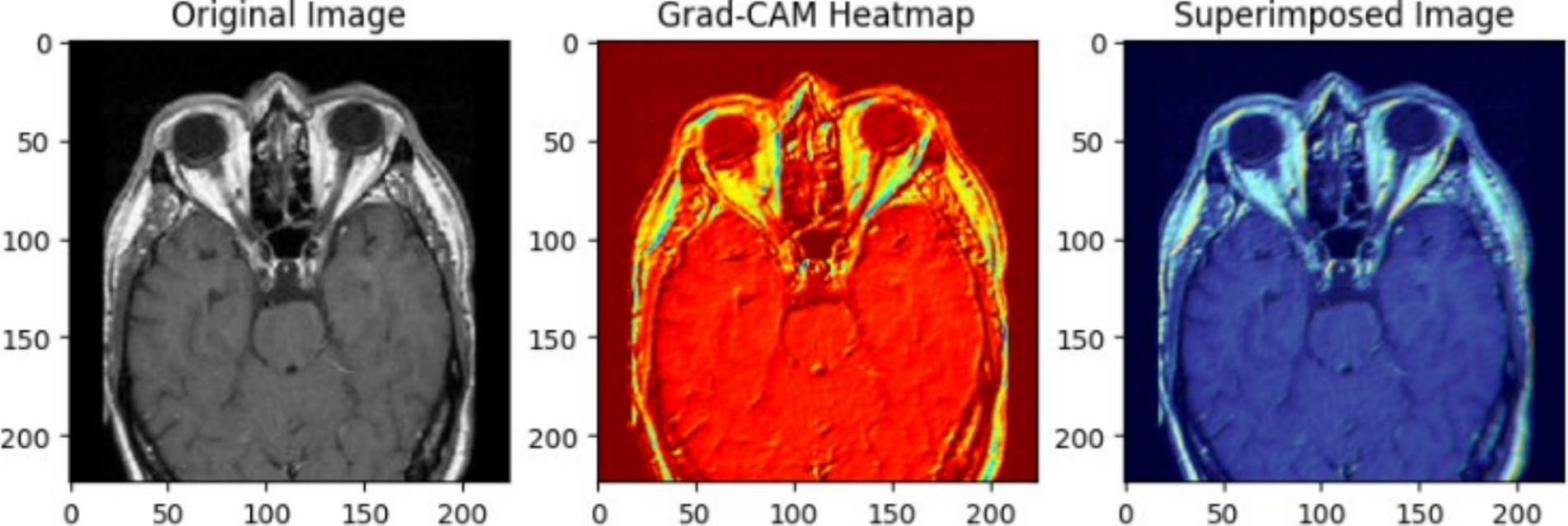
Grad-CAM heatmap visualization of normal MRI image in CNN layers.

In Figure 21, the Grad-CAM heatmap visualizes the regions of the brain MRI that contributes to the prediction of the model. The highlighted regions in red and yellow represents the regions with higher model attention, indicating potential tumor regions. By applying grad-cam in the early stages of the model development. it ensures that researchers can interpret how the model focuses on relevant anatomical structures instead of background details. This interpretability helps in refining the model architecture, adjusting hyperparameters, and understanding potential biases.

**Figure 22:**
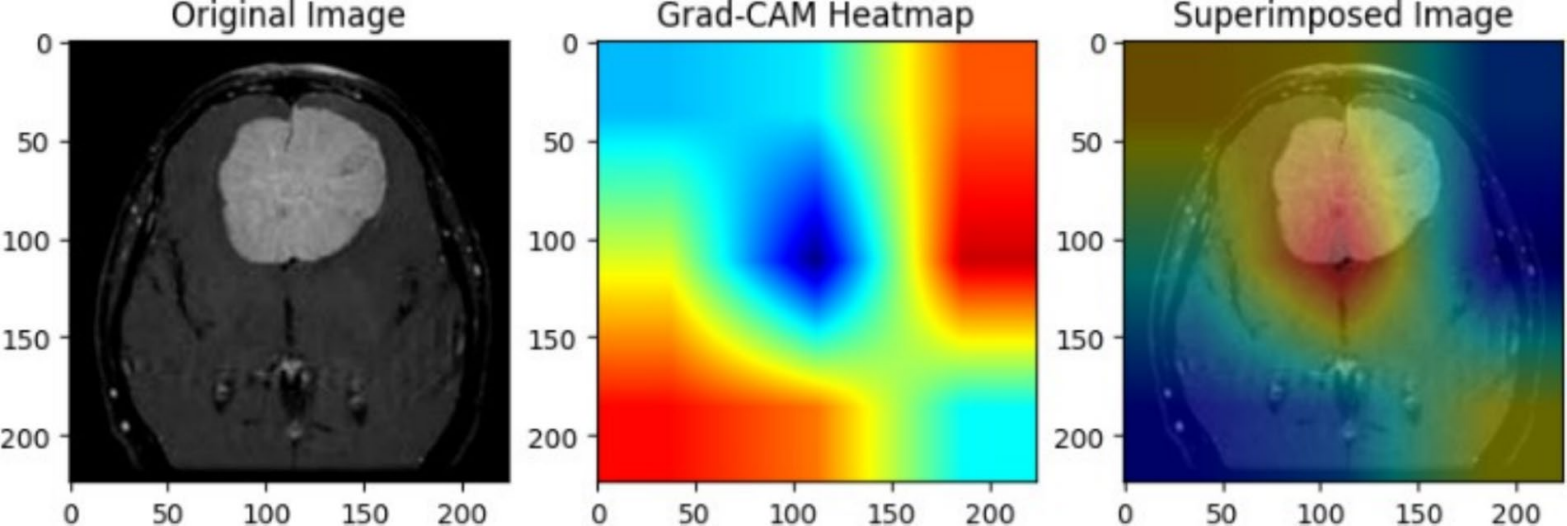
Grad-Cam heatmap visualization of glioma MRI image in Inceptionv3 layers.

However, there are limitations to these visualizations. As you can see in Figure 22, the Grad-Cam’s resolution is might often be coarse, meaning it may produce vague boundaries and in some cases, missing fine details. Additionally, Grad-CAM does not provide quantitative measure of uncertainty, which limits its decisions in cases where confidence assessment is necessary. Also, Grad-CAM is inherently limited by its dependency on convolutional layers, which makes it less effective for fully connected architectures.

On the other hand, LIME operates by perturbing the input image and analyzing the impact on predictions to approximate a locally interpretable linear model. The LIME results provided importance for the feature at the pixel level, offering a different perspective on model decision making. Unlike Grad-CAM, which highlights broad areas of interest, LIME produces superpixel-based explanations, indicating which specific regions, textures, or patterns influenced the classification. This feature is particularly beneficial in detecting false positives or understanding misclassifications, as LIME highlights not only tumor regions but also possible artifacts or non-relevant areas that affected the prediction.

**Figure 23:**
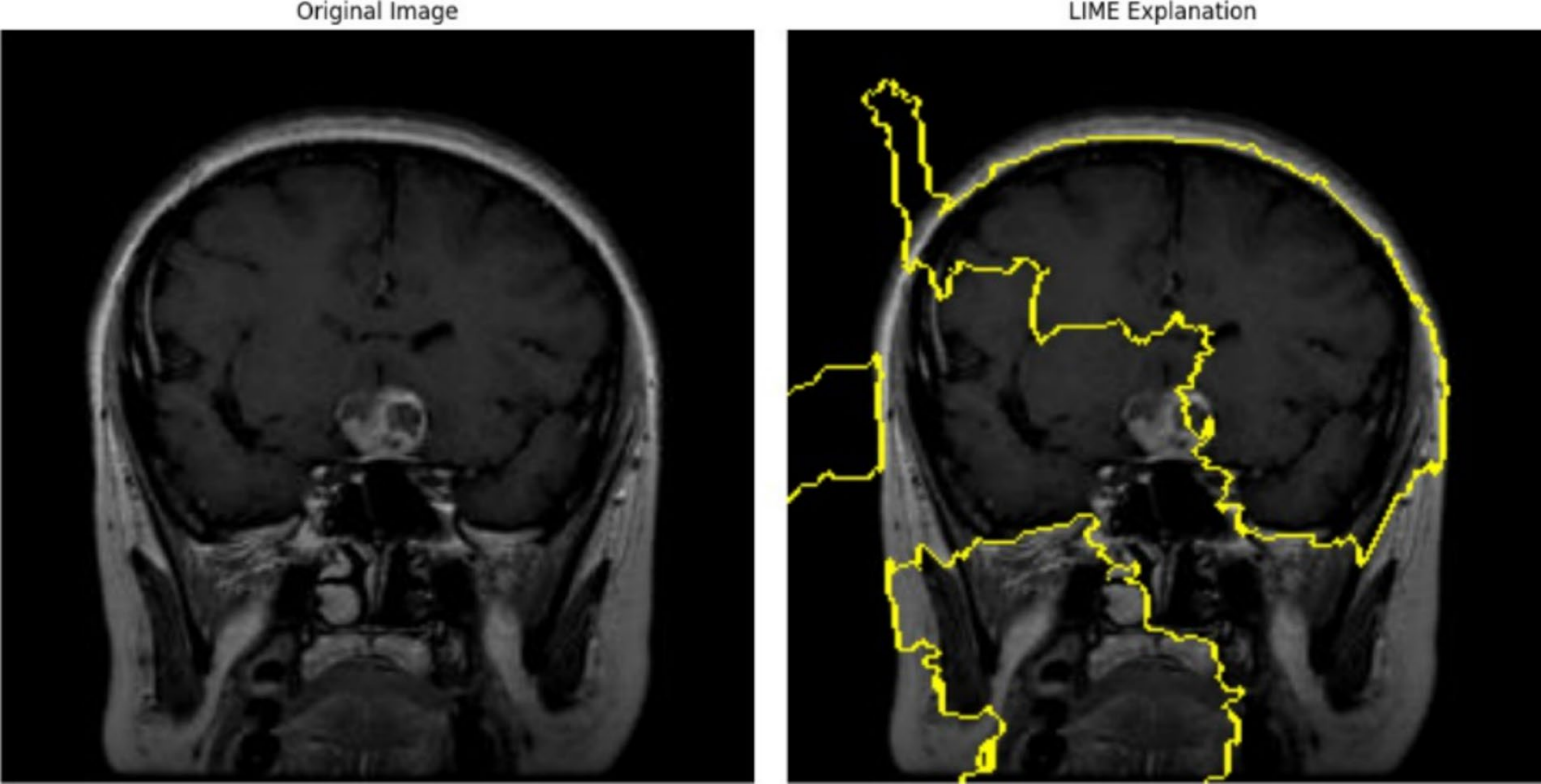
LIME visualization of pituitary MRI image in early stages of Xception model.

**Figure 24:**
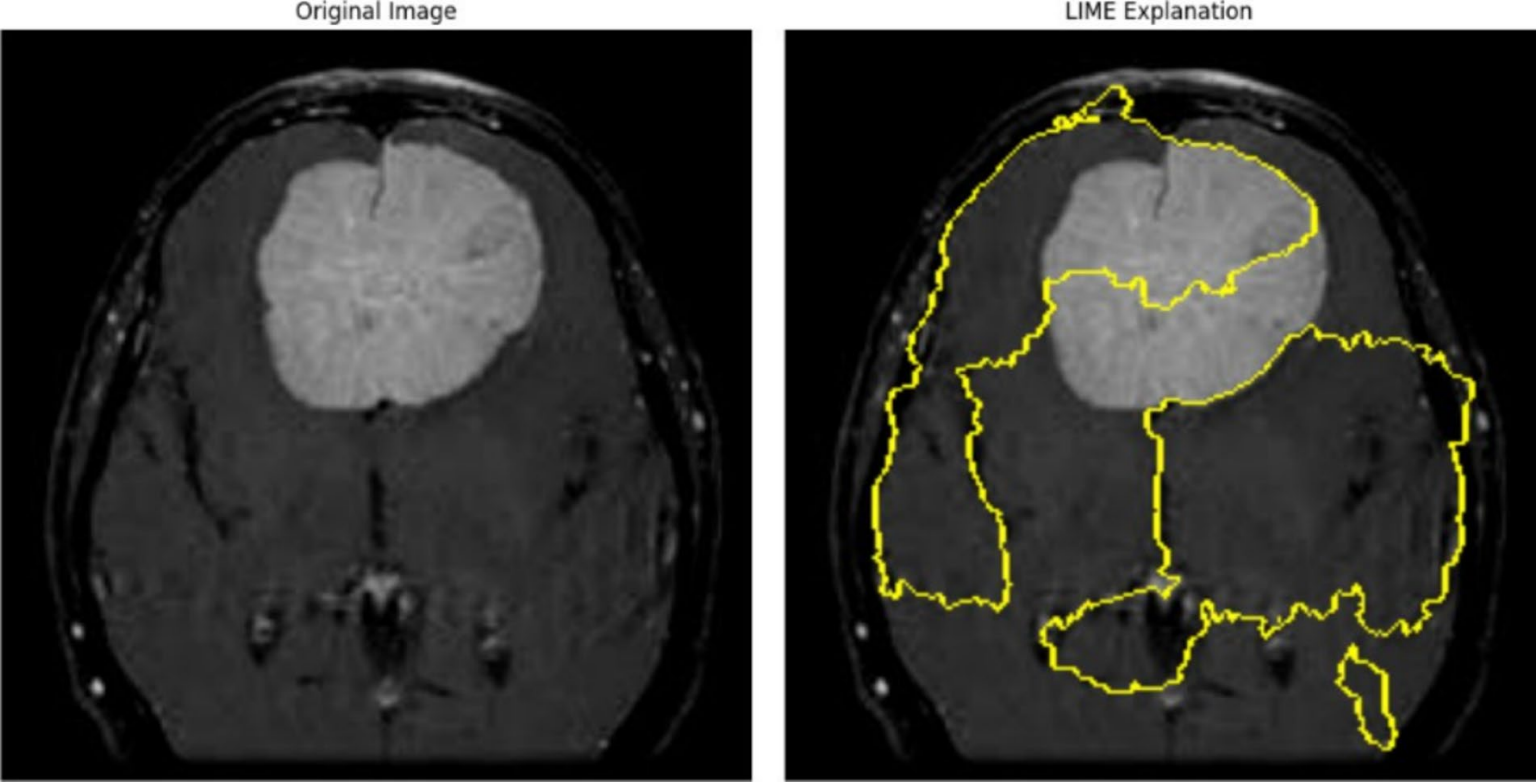
Lime visualization of glioma MRI image in densenet50 layers.

In Figure 23 and 24, the LIME visualizations highlight regions of the brain MRI that significantly contributed to the model’s decision. The highlighted yellow boundaries mark areas where the model focused its attention, correctly identifying the tumor. This local explanation helps validate the model’s accuracy and ensures it is not relying on irrelevant features. LIME is particularly useful in understanding how the model differentiates between healthy and abnormal tissue, providing a layer of interpretability often lacking in deep learning systems. However, LIME also has limitations. Consider Figures 19 and 20, it perturbs the input data and builds surrogate models to explain predictions, its explanations can vary based on the sampling process and parameter choices. Additionally, it may sometimes highlight non-tumor regions, especially in noisy or complex medical images. The localized nature of LIME explanations also means it might miss broader contextual insights that global interpretability methods could provide.

When comparing the two methods, Grad-CAM proves to be more effective for global interpretability, allowing domain experts to visualize the primary decision-making areas at a glance. This is crucial in confirming clinical validity, as a well-trained model should consistently focus on the tumor region across different images. LIME, in contrast, is more suitable for local interpretability, as it helps identify specific features and potential biases influencing the model’s classification. Its ability to provide fine-grained attributions makes it valuable for understanding misclassifications and edge cases in medical imaging.

Ultimately, the choice between Grad-CAM and LIME depends on the specific goals of the study. For validating model accuracy in medical diagnostics such as brain tumor classification, Grad-CAM offers a clear advantage. However, by combining both methods, XAI could offer a more comprehensive evaluation, leveraging Grad-CAM’s class-specific explanations and LIME’s broader interpretability.

### 6.7 Limitations

While the proposed approach demonstrates strong classification performance and interpretability, several limitations remain that warrant further investigation. First, the model was primarily trained on the Crystal Clean Brain Tumor MRI dataset sourced from Kaggle, which, despite its clarity and balanced labeling, may not fully capture the imaging diversity and clinical variability present in large-scale, multi-institutional datasets. Although cross-verification against other benchmark datasets indicated consistent performance, broader validation using heterogeneous datasets such as BraTS, TCIA, or local hospital archives is essential to confirm generalizability across scanners, acquisition protocols, and patient demographics.

Second, the explainability mechanisms employed—Grad-CAM and LIME—proved valuable in visualizing the model’s focus regions and refining architecture design. However, certain visual explanations occasionally highlighted non-tumor areas, suggesting either subtle biases in learned feature representations or inherent granularity limitations in current XAI methods. While the dataset annotations provided reliable tumor localization, further validation from radiologists and neuro-oncology experts would enhance confidence in these interpretability outputs and ensure that the visual reasoning aligns with clinical judgment.

Third, the current study was limited to classification and did not incorporate segmentation, which is essential for delineating tumor boundaries and guiding clinical decision-making such as surgical planning or radiotherapy targeting. Extending the framework to include joint segmentation–classification pipelines or multimodal MRI integration could offer a more holistic diagnostic tool. Additionally, computational scalability and real-time deployment remain challenges—especially for integration into federated or privacy-preserving frameworks, which could help address data-sharing constraints in clinical environments.

Overall, while the proposed XAI-integrated CNN framework offers an interpretable and efficient solution for brain tumor classification, future work should focus on multi-center validation, expert-based explanation assessment, and the inclusion of segmentation and federated extensions to strengthen clinical applicability and ethical transparency. Future clinical integration must also address ethical and privacy considerations. Future clinical integration must also address ethical and privacy considerations. Approaches such as Argumentation-based Explainable AI Caroprese et al. (2023) could support transparent reasoning and traceable decision pathways. Similarly, federated learning frameworks (Gagliardi et al., 2025) offer potential for distributed training across medical institutions, preserving patient confidentiality while enhancing dataset diversity.

## 7 Conclusion

The brain tumor classification project achieved its goal by developing and deploying deep learning models to accurately classify brain tumors from MRI data. Various architectures, such as Convolutional Neural Networks (CNNs) and Transformer-based models, were designed, trained, and evaluated for their effectiveness in handling medical imaging data. The CNN models excelled in feature extraction and classification tasks, reaching an accuracy of up to 98.65%, making them valuable tools for medical practitioners in detecting and diagnosing brain tumors. While Transformer models showed potential, they required more computational resources and fine-tuning to improve their efficiency for real-time deployment.

To ensure accessibility, the project deployed the best-performing models through web interfaces using frameworks like Streamlit and Gradio. These interfaces allowed for seamless real-time image uploads and classification, providing an intuitive experience for users. The deployment demonstrated the practical application of deep learning in medical diagnostics, supporting radiologists and healthcare professionals in identifying brain tumors early. The project highlights the potential of deep learning in medical image classification and lays the foundation for future advancements, evolving into a more powerful tool for early tumor detection and diagnosis. Looking ahead, there are areas for improvement and expansion in the brain tumor classification project.

Future work will involve refining advanced models with improved attention mechanisms and feature extraction techniques, enhancing the ability to detect subtle tumor features. An exciting prospect is the integration of Large Language Models (LLMs) after the classification stage, generating detailed diagnostic reports that summarize the findings and provide insights into tumor characteristics and recommended actions. This integration would enhance the interpretability and usefulness of the model’s output, bridging the gap between predictions and clinical insights.

Additionally, segmentation of brain tumors remains a key area for future development. Using the BraTS dataset will improve tumor boundary detection, aiding in assessing tumor growth and response to treatment. Future research will focus on developing segmentation models integrated with the classification models for comprehensive brain tumor analysis. Rigorous fine-tuning of classification models and LLMs will enhance accuracy, robustness, and generalization across various datasets and clinical scenarios. Exploring multimodal MRI data integration will capture diverse tumor characteristics, improving accuracy and comprehensiveness, providing a holistic view for better diagnosis and treatment planning. In summary, the current project has shown promising results in brain tumor classification, and future work will enhance the system’s capabilities, making it more accurate, accessible, and effective in clinical practice.

Although the proposed system demonstrates promising performance, several limitations should be acknowledged. First, the dataset used in this study may not fully capture the diversity of patient populations or imaging conditions encountered in real-world clinical settings. This may affect the model’s generalizability across different institutions and acquisition protocols. Second, the system’s performance has not yet been validated on external, multi-center datasets, which is essential to assess cross-site robustness. Finally, while the current implementation is computationally efficient for research-scale experiments, further optimization and integration with hospital information systems would be required for scalable clinical deployment.

## Data Availability

Dataset is openly available from the Kaggle, https://www.kaggle.com/datasets/mohammadhossein77/brain-tumors-dataset.

## Footnotes

1
https://www.kaggle.com/datasets/sartajbhuvaji/brain-tumor-classification-mri
